# Impact of photosensitizer orientation on the distance dependent photocatalytic activity in zinc phthalocyanine–nanoporous gold hybrid systems†

**DOI:** 10.1039/d0ra03891a

**Published:** 2020-06-17

**Authors:** David Steinebrunner, Günter Schnurpfeil, Mathis Kohröde, Alexander Epp, Khaetthariya Klangnog, Jorge Adrian Tapia Burgos, Andre Wichmann, Dieter Wöhrle, Arne Wittstock

**Affiliations:** Institute of Applied and Physical Chemistry and Center for Environmental Research and Sustainable Technology, University Bremen Leobener Str. UFT 28359 Bremen Germany awittstock@uni-bremen.de; MAPEX Center for Materials and Processes, University Bremen Bibliothekstr. 1 28359 Bremen Germany; Organic and Macromolecular Chemistry, University Bremen Leobener Str. NW2 28359 Bremen Germany woehrle@uni-bremen.de

## Abstract

Nanoporous gold powder was functionalized in a two-step approach by an azide terminated alkanethiol self-assembled monolayer (SAM) and a zinc(ii) phthalocyanine (ZnPc) derivative by copper catalyzed azide-alkyne cycloaddition (CuAAC). A series of different hybrid systems with systematic variation of the alkyl chain length on both positions, the alkanethiol SAM and the peripheral substituents of the ZnPc derivative, was prepared and studied in the photooxidation of diphenylisobenzofuran (DPBF). An enhancement by nearly one order of magnitude was observed for the photosensitized singlet oxygen (^1^O_2_) generation of the hybrid systems compared to the same amount of ZnPc in solution caused by the interaction of the npAu surface plasmon resonance and the excited state of the immobilized sensitizer. This interaction was shown to be distance dependent, with decreasing activity for short SAMs with alkyl chain lengths < 6 methylene groups caused by quenching of the excited state *via* electron transfer as well as decreasing activity for SAMs with *n* > 8 methylene groups due to decreasing energy transfer for long distances. An unexpected distance dependent behaviour was observed for the variation of the peripheral alkyl chain on the photosensitizer revealing a planar orientation of the immobilized photosensitizer on the nanoporous gold surface by a penta-coordinated central zinc ion through interaction with free azide groups from the self-assembled monolayer.

## Abstract 2

The distance dependency of the photocatalytic activity in zinc phthalocyanine–nanoporous gold hybrid systems was investigated revealing the importance of photosensitizer orientation in novel hybrid-based photocatalysts.

## Graphical abstract

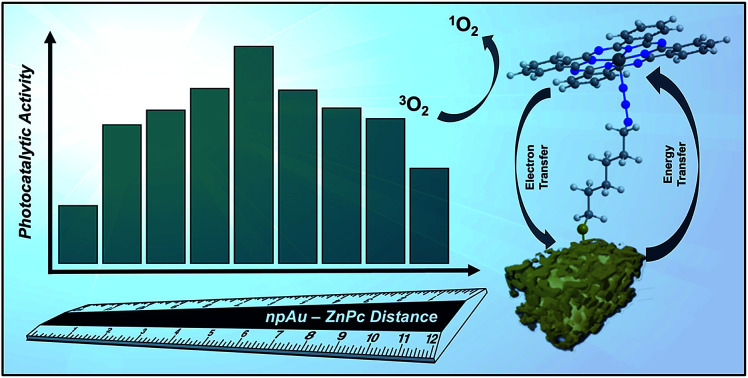

## Introduction

The field of hybrid materials consisting of a nanostructured metal and various organic or organometallic functionalities has recently gained growing attention due to their possible applications in different fields ranging from catalysis and sensor applications to new medical treatments.^1–4^ Especially regarding their photophysical properties those hybrid systems are of emerging interest as many of the used nanostructured metal skeletons show special optical properties due to their surface plasmon resonance.^4–6^ Therefore, several hybrid systems were designed and recently reported as alternatives for classical organometallic systems for example in photon upconverting systems or as completely new approaches like plasmonic sensing or medical applications like photodynamic cancer therapy (PDT).^7–12^ All of these processes are based on a photophysical interaction between the nanostructured metal support and the attached functionality, mostly in the form of energy transfer from the plasmon resonance to the excited state of the attached organic or organometallic moiety.^7,13,14^

The probably best understood type of hybrid system in this context is the one usually used for PDT consisting of either a metal phthalocyanine or porphyrin derivative attached to gold nanoparticles (AuNPs). Since their first report by Russell and coworkers in 2002, several studies on this type of hybrid materials were performed showing that such systems are superb ^1^O_2_ sensitizers with increased photocatalytic activities compared to the analogous complexes used in solution.^15,16^ The underlying photophysical interactions were investigated in detail in the group of Lemmetyinen, showing that various energy and electron transfer processes occur in such systems, which are also responsible for the observed increase in ^1^O_2_ formation.^13,17^

**Fig. 1:**
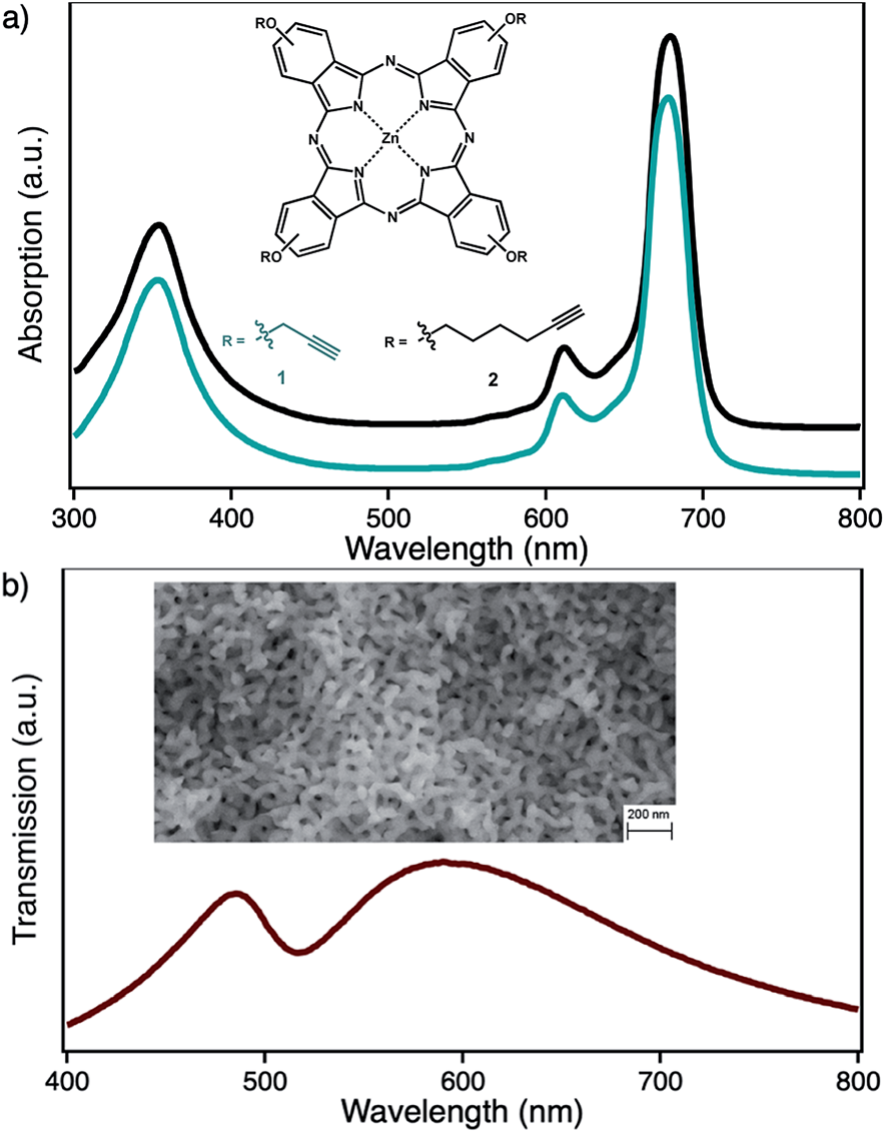
Structure and corresponding optical spectra for (a) the photosensitizer zinc(ii) phthalocyanine with either peripheral propynoxy- (1, cyan) or hexynoxy-substituents (2, black) and (b) nanoporous gold with pore sizes of 40 nm.

In the context of photocatalytical ^1^O_2_ based oxidation reactions, that are also of interest in industrial processes, colloidal nanoparticle systems are only of minor interest as truly heterogeneous systems are usually better in terms of lifetime and recyclability. Here, nanoporous gold (npAu, Fig. 1b) could be a valuable alternative, as it shows comparable photophysical properties of AuNPs while fulfilling the criteria of a truly heterogeneous, monolithic catalyst.^3,18–20^ This monolithic material consists of ligaments and pores on the order of 40 nm throughout macroscopic dimensions of up to centimeters (Fig. 1b, inset). In addition, npAu was already shown to be an efficient catalyst for oxidation reactions in both, gas and liquid phase systems.^21–24^ On the other hand, the same principles for functionalization of the npAu surface with organic compounds such as macrocyclic metal complexes can be applied as in the case of AuNPs.

We recently reported a comparable system where a ZnPc derivative was immobilized on different shaped npAu supports, as examples for a truly heterogeneous monolithic system, a heterogeneous powder catalyst as well as a photocatalytic coating.^25–27^ These new hybrid photocatalysts showed high overall ^1^O_2_ sensitization activities, where the activity was an order of magnitude above the activity of the same amount of the photosensitizer in solution.^26^

**Fig. 2:**
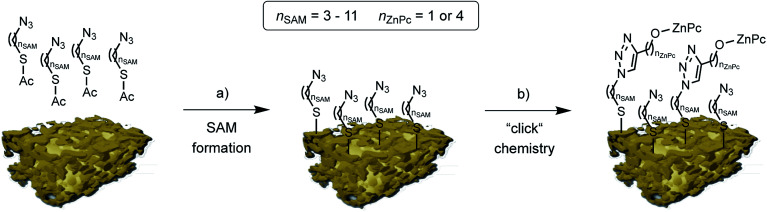
Schematic representation of the two step npAu powder functionalization: (a) formation of the azide terminated SAM using azidothioates with various alkyl chain lengths, and (b) copper catalyzed azide-alkyne cycloaddition (“click chemistry”) to bind the peripherally alkyne-substituted ZnPc derivatives 1 and 2. (npAu modified from [ref. 20]).

By selective irradiation of only the plasmon resonance of npAu (Fig. 1b) it was shown that a contribution by energy transfer to the attached ZnPc (Fig. 1a) is responsible for the enhanced activity.^26^ The investigation of the distance dependency of the energy transfer between both parts of the hybrid system is therefore of immense interest to optimize the system regarding its ^1^O_2_ sensitization activity. The system itself offers two different positions to achieve hybrid materials with several distances between the npAu surface and the attached photosensitizer. The variation of the alkyl chain length can be performed at the SAM alkanethiol as well as on the peripheral side chains of the ZnPc derivative (Fig. 2). The length of the alkyl chains, but also the orientation of the macrocycle on the surface contributes to the overall distance between the ZnPc and the npAu surface. Therefore, a careful investigation of both distance determining parameters in nanoscaled systems is essential as it can help to improve the design and understanding of novel hybrid systems in this recently strong and fast growing research field.

## Results and discussion

### Synthesis and characterization

The npAu powder support was prepared employing a free corrosion method of the starting alloy disk (Ag/Au, 70 : 30 at%) in concentrated HNO_3_ and powdering of the obtained npAu disk as described previously.^26^ The resulting powder showed a mean particle size of around 28 μm and pore sizes in the range of 40 nm.^26^ The residual silver concentration was below 1 at%, which was confirmed by EDX spectroscopy and shows good agreement to previously reported results.^28–32^

The organic functionalization of the npAu powder was achieved following a two-step approach established in our group (Fig. 2).^25–27^ In a first step, the surface of the npAu powder was functionalized with an azide-terminating SAM using acetyl-protected azidoalkylthioates. For the systematic variation of the distance between the immobilized photosensitizer and the npAu surface, SAMs with alkyl chain lengths ranging from *n*_SAM_ = 3 to 11 CH_2_ groups between the sulphur anchor and the free azide group were employed. In the following step, the respective ZnPc derivative with either a propynoxy- or hexynoxy-group with *n*_ZnPc_ = 1 or 4 CH_2_ groups as peripheral substituent was bound to the free azide groups of the SAM *via* copper catalyzed azide-alkyne cycloaddition (CuAAC). By this method a complete set of hybrid systems was obtained with chain lengths ranging from *n*_SAM_ = 3 to 11 CH_2_ groups in the basic SAM for both ZnPc derivatives (Table 1).

**Table 1:** Overview over the series of hybrid systems with systematic variation of the ZnPc–npAu distance and the corresponding immobilization fractions. Hybrid – abbreviation used in the text for the corresponding hybrid system, *n*_SAM_ – total number of CH_2_ groups in the alkylthiol SAM, *n*_ZnPc_ – number of CH_2_ groups in the peripheral substituent of the ZnPc photosensitizer, Zn [μg g_Catalyst_^−1^] – immobilized Zn content given as μg per g hybrid catalyst as obtained by ICP-MS measurements and ZnPc_Irr._ [mol] – determined photosensitizer amount immobilized on npAu and irradiated during the photocatalytic studies

Hybrid	*n*_SAM_	*n*_ZnPc_	Zn [μg g_Catalyst_^−1^]	ZnPc_Irr._ [mol]
H1-3	3	1	154.9	1.4 × 10^−10^
H2-3	3	4	151.7	1.4 × 10^−10^
H1-4	4	1	235.3	2.1 × 10^−10^
H2-4	4	4	191.1	1.8 × 10^−10^
H1-5	5	1	161.2	1.5 × 10^−10^
H2-5	5	4	165.9	1.5 × 10^−10^
H1-6	6	1	165.8	1.5 × 10^−10^
H2-6	6	4	189.6	1.7 × 10^−10^
H1-7	7	1	170.1	1.6 × 10^−10^
H2-7	7	4	150.0	1.4 × 10^−10^
H1-8	8	1	147.7	1.3 × 10^−10^
H2-8	8	4	133.4	1.2 × 10^−10^
H1-9	9	1	155.9	1.4 × 10^−10^
H2-9	9	4	187.6	1.7 × 10^−10^
H1-10	10	1	151.6	1.4 × 10^−10^
H2-10	10	4	176.1	1.6 × 10^−10^
H1-11	11	1	157.9	1.5 × 10^−10^
H2-11	11	4	183.9	1.7 × 10^−10^

Each of the obtained hybrid systems was analyzed in detail regarding its chemical composition and porous structure of the support. It was shown by SEM that the porous structure of the npAu powder stayed unchanged at 40 nm during the functionalization. The energy dispersive X-ray spectroscopy (EDX) measurements of all samples showed a homogeneous zinc distribution, which was confirmed by EDX mapping and line scan experiments. This confirms previous results of selected systems as described previously by us.^26,27^

The amount of immobilized sensitizer in every hybrid system was determined by quantification of the amount of Zn in the system by inductively coupled plasma mass spectrometry (ICP-MS) after dissolution of the corresponding sample in ultrapure *aqua regia*. The obtained values as well as the light exposed fractions of the immobilized sensitizer are summarized in Table 1. The amount of light exposed sensitizer was around 1.5 × 10^−10^ mol for every hybrid system which was determined from the immobilized sensitizer amount and a penetration depth of 300 nm for visible light into the nanoporous structure.^26^

### Distance dependent photocatalytic activity

**Fig. 3:**
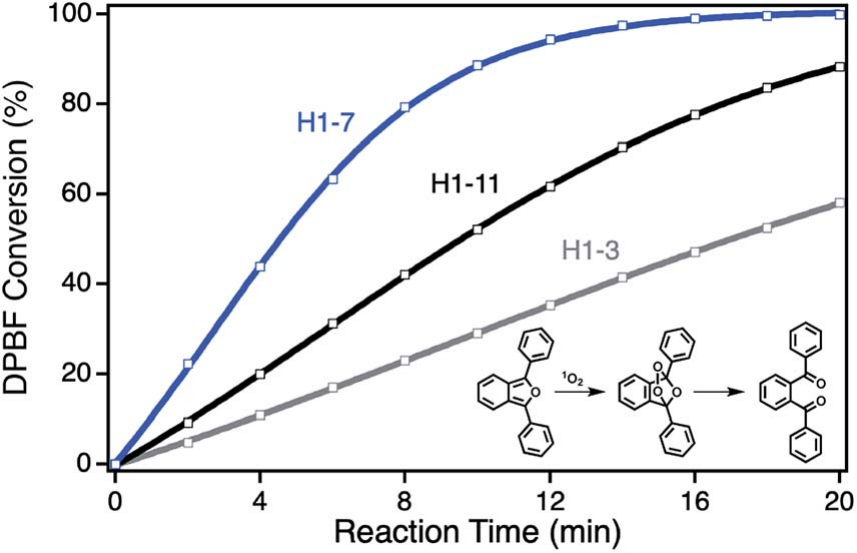
DPBF conversion curves exemplarily showing the activity for a short SAM (H1-3, gray) with strong quenching by electron transfer from ZnPc to npAu, a medium SAM (H1-7, blue) with highest energy transfer from npAu to ZnPc and a long SAM (H1-11, black) with decreasing energy transfer from npAu to ZnPc. Inset: reaction scheme for the photooxidation of DBPF used for the determination of ^1^O_2_ production as selective chemical quencher.

For the determination of the photocatalytic activity of each ZnPc–npAu hybrid system, the photocatalysts were studied in the photooxidation reaction of 1,3-diphenylisobenzofuran (DPBF). DPBF is the most used chemical quencher for singlet oxygen and reacts selectively and almost quantitatively *via* an endoperoxide to 1,2-dibenzoylbenzene (Fig. 3, inset). The photooxidation reactions were conducted in a self-built photocatalytic setup described previously under irradiation with a 550 nm cut-on filter, 700 nm bandpass or 550 nm bandpass filter to achieve selective irradiation of the immobilized ZnPc, the npAu plasmon resonance or both active sites simultaneously.^26,27^ The decrease of DPBF was followed by UV-Vis spectroscopy at *λ* = 415 nm and the photooxidations were quantified by their corresponding turnover numbers (TON) and turnover frequencies (TOF, Fig. S1–S18†).

**Fig. 4:**
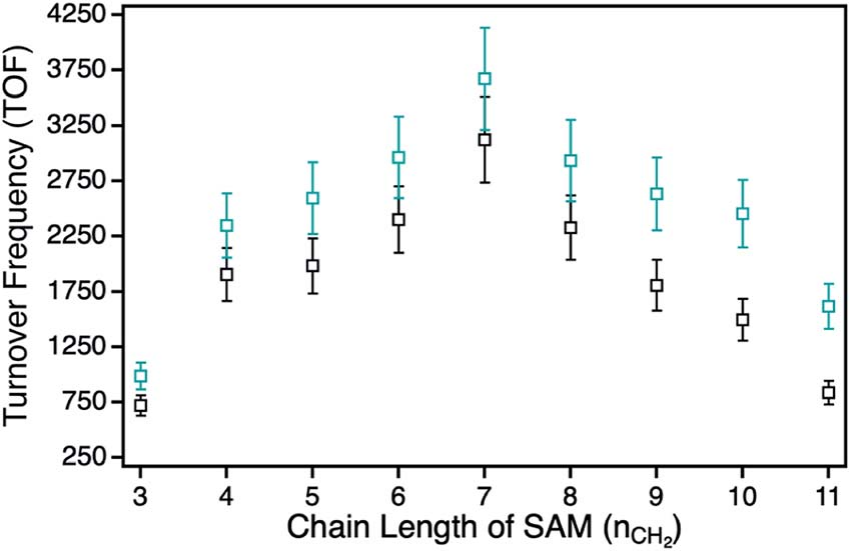
Photocatalytic ^1^O_2_ sensitization activities for the hybrids with a systematic variation of the alkyl chain lengths plotted as function of the SAM chain length. Series H1-3–H1-11 with the peripheral propynoxy-functionalized ZnPc derivative 1 is the cyan trace and series H2-3–H2-11 with peripheral hexynoxy-functionalities (ZnPc 2) the black trace.

For the ZnPc derivative 1 with peripheral propynoxy-substituents the TOF values showed a clear trend with maximum activity for the hybrid H1-7 build of a SAM with seven methylene groups. In contrast, for longer and shorter distances the photocatalytic singlet oxygen sensitization activity decreases rapidly (Table 2, Fig. 3 and 4). Similar trends were observed with decreasing photocatalytic activities by irradiation of mainly the ZnPc using a 700 nm bandpass filter. A further decrease was observed for irradiation of only the plasmon resonance of npAu employing a 550 nm bandpass filter. The highest activities using the 550 nm cut-on filter clearly shows the synergistic effect of the npAu surface plasmons for the generation of singlet oxygen (Table 2).

**Table 2:** Summarized photocatalytic activities determined for the two series of hybrid systems with systematic variation of the ZnPc–npAu distance under irradiation with a 550 nm cut-on (TOF_co_), 700 nm bandpass (TOF_700_) and 550 nm bandpass (TOF_550_) filter. *d*_SAM_ [nm] – calculated thickness of the SAM showing the distance between the npAu surface and the terminal N atom of the azide groups of the SAM with either a short (s) or long (l) axial coordination distance to the central Zn ion of the immobilized ZnPc derivative

Hybrid	TOF_co_ [min^−1^]	TOF_700_ [min^−1^]	TOF_550_ [min^−1^]	*d*_SAM_ [nm]
H1-3	988.9	639.7	99.2	0.96 (s)
H2-3	719.6	428.3	60.6	0.96 (l)
H1-4	2347.8	1729.1	186.8	1.07 (s)
H2-4	1905.7	975.8	127.8	1.07 (l)
H1-5	2596.6	1979.4	243.9	1.21 (s)
H2-5	1984.8	1070.4	136.9	1.21 (l)
H1-6	2961.9	2164.9	294.4	1.32 (s)
H2-6	2400.9	1308.8	171.2	1.32 (l)
H1-7	3670.8	2481.8	304.3	1.46 (s)
H2-7	3125.1	1826.1	260.7	1.46 (l)
H1-8	2934.9	1872.9	254.1	1.58 (s)
H2-8	2329.8	1503.2	187.2	1.58 (l)
H1-9	2634.1	1624.7	238.3	1.71 (s)
H2-9	1807.9	1316.7	149.4	1.71 (l)
H1-10	2452.6	1601.6	171.2	1.83 (s)
H2-10	1497.8	1026.8	119.7	1.83 (l)
H1-11	1619.4	949.1	119.5	1.96 (s)
H2-11	835.9	552.3	53.5	1.96 (l)

For the photophysical interactions, two different pathways are possible in such hybrid materials, which were shown spectroscopically in detail on AuNP systems. Lemmetyinen and coworkers observed energy transfer from both, the excited ZnPc to the AuNPs and *vice versa*.^13,17^ Also, electron transfer from the ZnPc to the AuNPs resulting in the corresponding phthalocyanine radical cation was observed.^13^ In general, energy transfer is a long ranging interaction with decreasing efficiency from 1 to 10 nm distance, whereas electron transfer is usually limited by a distance between donor and acceptor of around 1–2 nm in alkanethiol-bridged systems.^33,34^ The decreasing singlet oxygen sensitization activity for the hybrid systems H1-3 to H1-6 is therefore a result of the competition between energy and electron transfer, where electron transfer for shorter distances gets predominant. This process acts as quenching of the excited state of the ZnPc photosensitizer with negative influence on the singlet oxygen formation. For the hybrid systems H1-7 to H1-11 the distance between donor and acceptor is beyond the electron transfer regime, and therefore only energy transfer takes place with decreasing activity towards larger distances.

The series of the ZnPc derivative 2 with peripheral hexynoxy-substituents surprisingly showed a similar trend as the series of the ZnPc derivative 1 with the maximum activity for the hybrid H2-7. The activities for all other hybrids of this series showed the same electron and energy transfer behaviour for longer and shorter chain lengths of the SAM as already discussed for the first series. This observation is quite unexpected as, for an example, H1-6 and H2-3 should have the same distance between the immobilized sensitizer and the npAu surface due to the same number of CH_2_ groups if the linking unit is fully linearly extended. Therefore H1-6 and H2-3 should show a comparable photocatalytic activity. But in fact, the observed activities exhibit a completely contrary result as H2-3 shows the lowest activity of all hybrid systems whereas H1-6 is one of the most active hybrids.

The same distance dependent behaviour for both ZnPc series shows unambiguously that the overall distance between the two active centers is mainly determined by the chain length of the employed azidoalkylthioate used for the SAM formation. This fact is indeed contrary to a fully linearly extended orientation of the immobilized sensitizer and therefore a more detailed discussion about the orientation of the ZnPc macrocycle on the npAu surface is essential to understand the observed photocatalytic activities.

### Orientation of the photosensitizer on the surface

The distance between the immobilized photosensitizer and the npAu support is determined by the chain length of the employed SAM, but at a certain degree also by the orientation of the photosensitizer on the surface. Taking two possible orientations into account, namely a fully extended perpendicular orientation in respect to the support, as well as a planar, parallel orientation, the distance between the central Zn ion and the npAu surface shows a significant deviation. Whereas, for example, system H2-6 in planar configuration shows a separation distance of around 1.3 nm, the distance between the Zn ion and the npAu surface enlarges to nearly 1.9 nm for a perpendicular orientation.

The nature of the tetra peripheral substituted ZnPc derivative indeed can easily adopt such a planar configuration by undergoing multiple CuAAC reactions with up to four binding sites. There are some studies about the orientation of phthalocyanines and closely related macrocycles like porphyrins and porphyrazines on Au surfaces reported in literature, but all of them were prepared employing thiol-containing derivatives as precursors for SAM formation and none of those systems was prepared using the CuAAC reaction.^35–40^ Therefore, although for some systems a planar orientation is reported, the hybrid system reported here may vary in this respect due to the different preparation method. In addition, all of these experiments were performed on single crystal surfaces and therefore those results, but also most of the analysis techniques, are not directly transferable to a nanoporous surface.

**Fig. 5:**
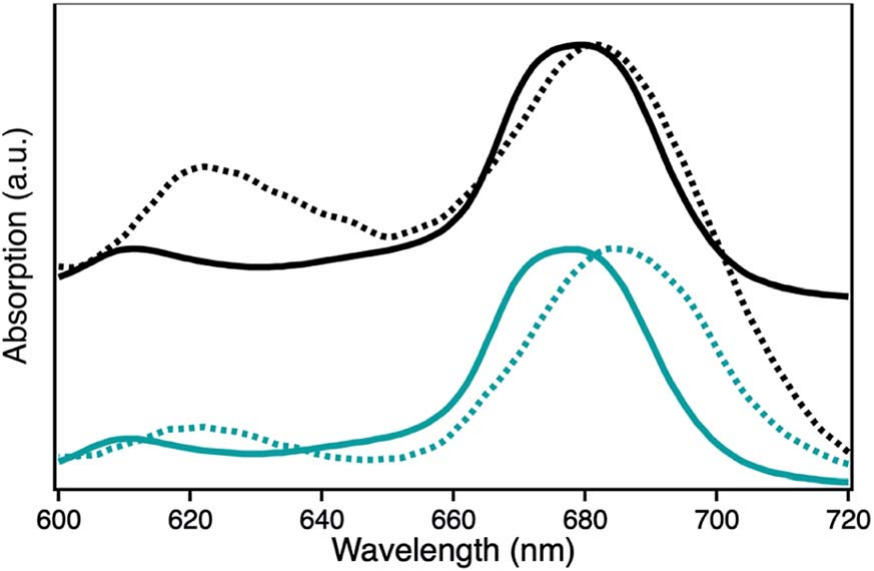
UV-Vis spectra showing the absorption of the Q-band of the photosensitizer ZnPc with either peripheral propynoxy- (1, H1-7, cyan) or hexynoxy-substituents (2, H2-7, black) in solution (bold line) and when immobilized on a npAu foil (dashed line).

The UV-Vis spectra of H1-7 and H2-7 when prepared on a light penetrable, 100 nm thick npAu foil exhibit an interesting feature where the absorption of the ZnPc Q-band is red-shifted compared to the absorption of the same sensitizer in solution, both times measured in a DMF environment (Fig. 5). Such a redshift of the Q-band of phthalocyanines and porphyrins is known to be caused by axial coordination of a nitrogen atom from an organic amine.^41–44^ The binding of an additional ligand in axial position is a common feature of zinc phthalocyanines and porphyrins derivatives, even forming stable crystal structures, supramolecular dimers and trimers as well as hybrid systems directly immobilized on amine-terminated SAMs.^45–53^ In addition, the penta-coordinating ability of the Zn central ion of zinc porphyrins is used frequently as template in many supramolecular synthesis approaches.^54–57^

**Fig. 6:**
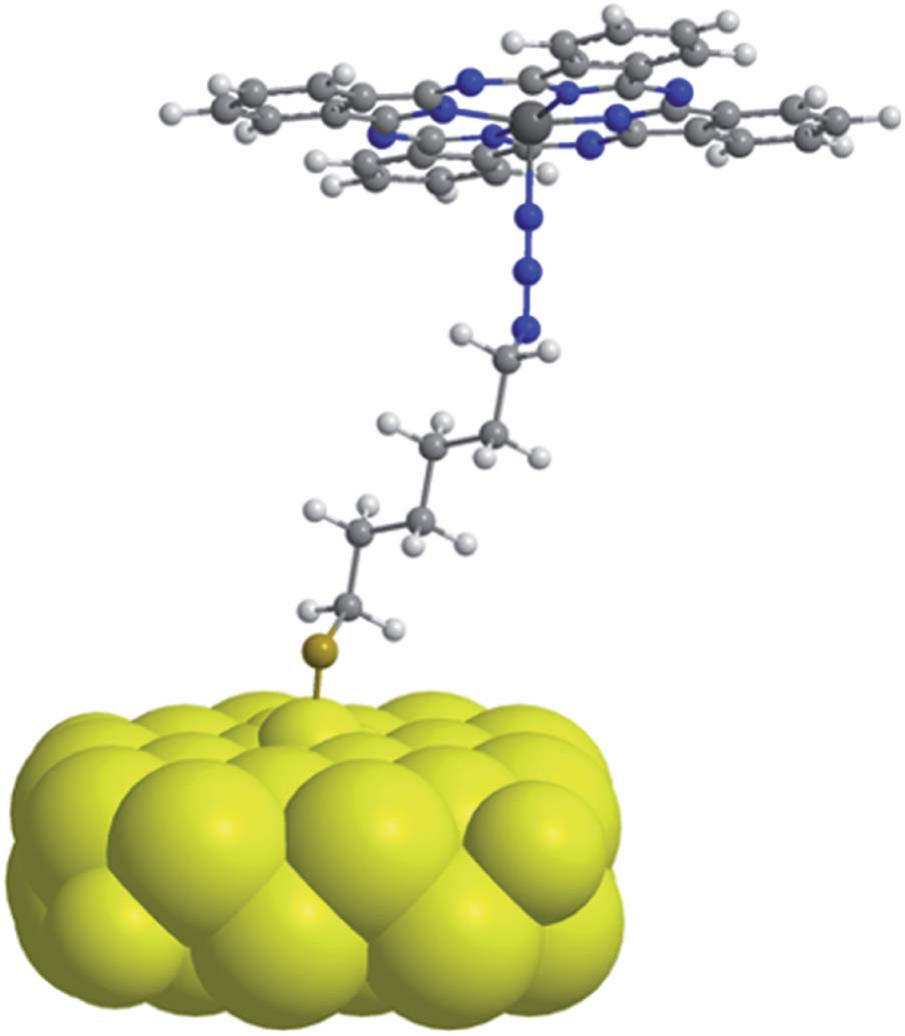
Simplified schematic illustration of the axial coordination between an immobilized ZnPc molecule on the npAu surface representing the dominant pathway for photoinduced interactions in the ZnPc–npAu hybrid systems. Peripheral substituents of the ZnPc forming covalent connections to the npAu surface through CuAAC were omitted for clarity.

The observed redshift after immobilization of the ZnPc onto the npAu surface suggests a similar penta-coordinated central ion, which is possible by interactions with free electron pairs of the azide groups from the SAM, representing the main pathway for photoinduced interactions in the hybrid systems (Fig. 6). Such an expected interaction might be even forced when the tetrasubstituted photosensitizer undergoes two or more triazole-based connections to the SAM, bringing the Zn central ion in a template-like direction to the free azide groups of the SAM. Such a coordination mode would result in a distance between the immobilized ZnPc and the npAu surface, which is determined by the chain length of the SAM as well as the axial coordination strength and can explain the observed distance dependent photocatalytic behaviour of the hybrid systems. The slightly higher activity that was found for the series of the ZnPc derivative bearing propynoxy-substituents can be explained by a stronger interaction of the Zn ion with the azide groups compared to the hexynoxy-substituted ZnPc derivative due to the shorter peripheral substituent as indicated by the different shifts of the Q-band after immobilization (Fig. 5).

Another interesting point is the position of the maximum photocatalytic activity for the hybrid systems with *n*_SAM_ = 7 methylene groups in the SAM. By comparison of the photocatalytic activities and the calculated distances between the Zn center and the npAu surface (Table 2), the maximum was found for a distance around 1.5 nm. This finding is also important to that extend that a distance slightly above 1 nm is often described as the border until electron transfer is possible through alkanethiol-based bridges and only energy transfer is observed for further increasing distances.^33,34^ In hybrid systems of pyrene-functionalized AuNPs with different alkylthiol linkers, photoinduced electron transfer to the AuNP was observed in 5 methylene containing bridges but not for similar systems containing bridges of 8 methylene groups.^33^ Therefore, the observed maximum activity around 1.5 nm also supports our suggested planar configuration and our hypothesis of an interaction with the free azide groups.

In previous studies, the coverage of the npAu surface was found to be around 1% of a monolayer, calculated on the assumption that a complete monolayer is based on a 1 : 1 ratio of immobilized photosensitizer molecules to available Au surface atoms. Considering the planar orientation of the photosensitizer on the surface as well as the van der Waals radius of gold, it is obvious that this assumption cannot represent the steric demand of a ZnPc molecule. As a single ZnPc core without any attached peripheral substituents already covers an area of at least 25 gold atoms when adopting a planar orientation on the Au surface, a value of 1% of the previous assumption would correspond to a value of at least 25% of a ZnPc monolayer on the Au surface.

In general, it should be mentioned that the “click-methodology”, namely the CuAAC reaction, is a convenient and versatile method often used for the functionalization of nanostructured surfaces. The attachment of photosensitizers, especially of phthalocyanines or porphyrins derivatives, employing this method is a quite common synthesis strategy for the preparation of various hybrid materials.^58–62^ Although used frequently, the characterization of as prepared hybrid materials is often challenging, which might be the reason that little is known about the orientation of the macromolecules in such hybrid systems. However, especially when investigating photoinduced interactions, the orientation can have a crucial impact on the overall donor–acceptor distance and therefore possible interactions between immobilized sensitizers and free functional groups of the SAM need to be taken into consideration and discussed properly in the context of new hybrid materials.

## Conclusions

In this study the distance dependent interactions in hybrid systems based on zinc(ii) phthalocyanine derivatives immobilized on a npAu support were studied. For this purpose, a systematic variation of the distance between the two chromophores was performed by variation of the alkyl chain length within the SAM as well as on the peripheral substituents on the ZnPc derivative. The whole series of hybrid systems was tested in the photooxidation of DPBF as standard reaction for singlet oxygen sensitizing systems.

The distance dependent photocatalytic activities, the low concentration of the immobilized photosensitizer as well as the redshift of the Q-band all supports the suggested planar orientation of the phthalocyanine ring with respect to the npAu surface, also providing evidence for a penta-coordinated Zn central ion interacting with the terminal azide groups of the SAM. The orientation of the sensitizer as consequence of the chosen preparation strategy using the established CuAAC reaction is an important step towards a better understanding of photoinduced interactions in hybrid systems as well as a template tool for the design and synthesis of novel hybrid-based materials.

## Experimental

### Materials

Alloy disks with Ag/Au ratio of 70 : 30 at% were prepared within our group according to a previously published procedure.^63^ American white gold (12 karat, Ag/Au 50 : 50 wt%) as starting alloy for npAu foils was bought at Noris Blattgold GmbH, Schwabach, Germany. For the CuAAC, the catalyst Cu(MeCN)_4_PF_6_ (97%) from Aldrich, hydroquinone from Merck and the co-catalyst tris(benzyl-triazolylmethyl)amine (TBTA), which was obtained by a literature procedure,^64,65^ were used. The chemical ^1^O_2_ quencher 1,3-diphenylisobenzofuran (DPBF, >95%) was received from TCI. All solvents including ethanol (abs., reagent grade), THF (reagent grade, ≥99.0%), DMF (analytical reagent grade, ≥99.5%), HNO_3_ (analytical reagent grade, 65% and NORMATOM, ultra-pure for trace analysis, 67%) and HCl (NORMATOM, ultra-pure for trace analysis, 34%) were bought at VWR and were used as received without further purification.

### Synthesis of the ZnPc derivatives

2,9,16,23-Tetrakis(2-propyn-1-yloxy)phthalocyanine zinc(ii) (1)^66^ and 2,9,16,23-tetrakis(5-hexyn-1-yloxy)phthalocyanine zinc(ii) (2)^25^ were synthesized by cyclotetramerization of 4-(2-propynyloxy)-phthalonitrile or 7-(2-hexynyloxy)-phthalonitrile respectively with Zn(CH_3_COO)_2_·2H_2_O as reported previously (Scheme S1†).

### Synthesis of azidoalkylthioacetates

3-Azidopropyl-1-thioacetate (3),^67–69^ 4-azidobutyl-1-thioacetate (4),^69,70^ 5-azidopentyl-1-thioacetate (5),^71–73^ 6-azidohexyl-1-thioacetate (6),^73–75^ 7-azidoheptyl-1-thioacetate (7),^71,73,76^ 8-azidooctyl-1-thioacetate (8),^77–79^ 9-azidononyl-1-thioacetate (9),^79–81^ 10-azidodecyl-1-thioacetate (10)^81–83^ and 11-azidoundecyl-1-thioacetate (11)^25^ were all synthesized starting from the corresponding *n*-bromo-1-alkanol *via n*-azidoalkan-1-ol and *n*-azidoalkyl methanesulfonate according to literature procedures (Scheme S2†).

### Preparation of nanoporous gold

npAu powder was prepared according to a previously published procedure by dealloying of Ag/Au disks (70 : 30 at%, 5 mm diameter, 200 μm thickness) in concentrated HNO_3_ for 72 h and cleaning with H_2_O. Subsequently after dealloying the npAu disks were powdered by fragmentation with a metal pincers.^26^ Obtained npAu samples exhibit a mean pore size of 40 nm as determined by SEM measurements with a specific surface area of around 5 m^2^ g^−1^ as calculated according to a literature procedure and also shown by BET measurements on comparable npAu samples.^27,84,85^

npAu foils with comparable pore sizes were prepared by floating American white gold leaf (Ag/Au 50 : 50 wt%, 1.5 × 1.5 cm, 100 nm thickness) on concentrated HNO_3_ for 8 h, followed by floating on H_2_O according to a literature procedure.^27^

### Hybrid preparation

Following a general procedure,^26^ SAMs with different chain lengths were prepared by covering the npAu powder with an ethanolic solution (5 mL) of azidoalkylthioacetate (3–11, 1 mmol) for 72 h. After thoroughly washing with EtOH, the as prepared npAu samples were covered with a solution of the ZnPc derivative (1–2, 0.15 μmol), Cu(MeCN)_4_PF_6_ (931.8 μg, 2.5 μmmol), TBTA (1.326 mg, 2.5 μmol) and hydroquinone (272.5 μg, 2.5 μmmol), dissolved in a THF/H_2_O mixture (5 mL, 3 : 1 v%) for another 72 h. Afterwards, the as prepared hybrids were repeatedly washed with THF in order to remove any physisorbed material.

Hybrid systems for the UV-Vis measurements on npAu foils were prepared following the same procedure with slightly modified conditions as described recently for the preparation of photocatalytic coatings.^27^

### Characterization methods

The quantity of immobilized ZnPc on npAu was determined by inductively coupled-plasma mass spectrometry (ICP-MS, iCAP Q, Thermo Fisher Scientific GmbH) after dissolving 10 mg of the as prepared hybrid sample in ultrapure aqua regia (2 mL).

The pore size of the npAu support was determined acquiring micrographs of the samples on a scanning electron microscope (SEM, Supra 40, Zeiss, Germany) operated at 15.0 kV acceleration voltage, 300 pA probe current and 10 mm working distance. Characterization was performed by measuring the diameter of at least 250 pores in the obtained SEM images using the program ImageJ.

The zinc distribution on the surface was determined by energy dispersive X-ray spectroscopy (EDX, Bruker XFlash 6/30).

All UV-Vis spectra were recorded using a UV-1600PC UV-Vis spectrometer from VWR.

### Photocatalytic oxidation

Photocatalytic oxidations were carried out by irradiation of the npAu hybrid photocatalysts with a 300 W Xe-arc lamp in a self-built setup, as described elsewhere.^26^ Briefly, for each run the reaction vessel was filled with DMF (100 mL), the powder catalyst was added and the chamber flushed with O_2_ for 10 min to achieve gas saturation of the solvent. Prior to irradiation, a DMF solution (500 μL) containing DPBF (1.35 mg, 5 μmol) was added. The reaction progress was followed *via* UV-Vis spectroscopy, determining the decrease of the DPBF concentration using Lambert Beers law at the absorption maximum at 415 nm (*ε*_415_ = 23 000 L mol^−1^ cm^−1^ in DMF).^86^ Irradiation was carried out employing a 550 nm cut-on, a 700 nm bandpass or a 550 nm bandpass filter respectively. Photocatalytic activities were determined according to literature for immobilized sensitizers by determination of the turnover number (TON, [converted DPBF] (mol)/[irradiated ZnPc] (mol)) and turnover frequency (TOF, slope of the plot of TON *versus* reaction time (min^−1^)) for every hybrid system.^87^

### Computational methods

For the semi-empirical calculations, the Hyperchem 7.1 program was used. All ground-state compounds were optimized by the PM3 method (the Polak–Ribiere Algorithm and the RMS gradient of 0.001 kcal Å^−1^ mol^−1^). The following parameters were used: total charge: 0; spin multiplicity: 1; spin pairing: RHF; state: lowest; convergence limit: 0.00001.

## Conflicts of interest

There are no conflicts of interest to declare.

## Supplementary Material

RA-010-D0RA03891A-s001
