# Supplementary material for: Impact of photosensitizer orientation on the distance dependent photocatalytic activity in zinc phthalocyanine–nanoporous gold hybrid systems
Source: RSC Adv. 2020 Jun 17;10(39):23203–11. doi: 10.1039/d0ra03891a (PMC9054629; doi:10.1039/d0ra03891a)
Supplement: RA-010-D0RA03891A-s001 [file RA-010-D0RA03891A-s001.pdf]

Electronic Supplementary Material (ESI) for RSC Advances.
This journal is © The Royal Society of Chemistry 2020

Supporting Information

Impact of photosensitizer orientation on the distance dependent photocatalytic activity in zinc phthalocyanine – nanoporous gold hybrid systems

David Steinebrunner,[ab] Günter Schnurpfeil,[c] Mathis Kohröde,[a] Alexander Epp,[a] Khaetthariya Klangnog,[c] Jorge Adrian Tapia Burgos,[ab] Andre Wichmann,[a] Dieter Wöhrle[*c] and Arne Wittstock[*ab]

[a] *Institute of Applied and Physical Chemistry and Center for Environmental Research and Sustainable Technology, University Bremen, Leobener Str. UFT, 28359 Bremen, Germany.*

[b] *MAPEX Center for Materials and Processes, University Bremen, Bibliothekstr. 1, 28359 Bremen, Germany.*

[c] *Organic and Macromolecular Chemistry, University Bremen, Leobener Str. NW2, 28359 Bremen, Germany.*

ESI-1 Supplementary data for the photooxidation of DPBF with H1-3 using different irradiation wavelengths

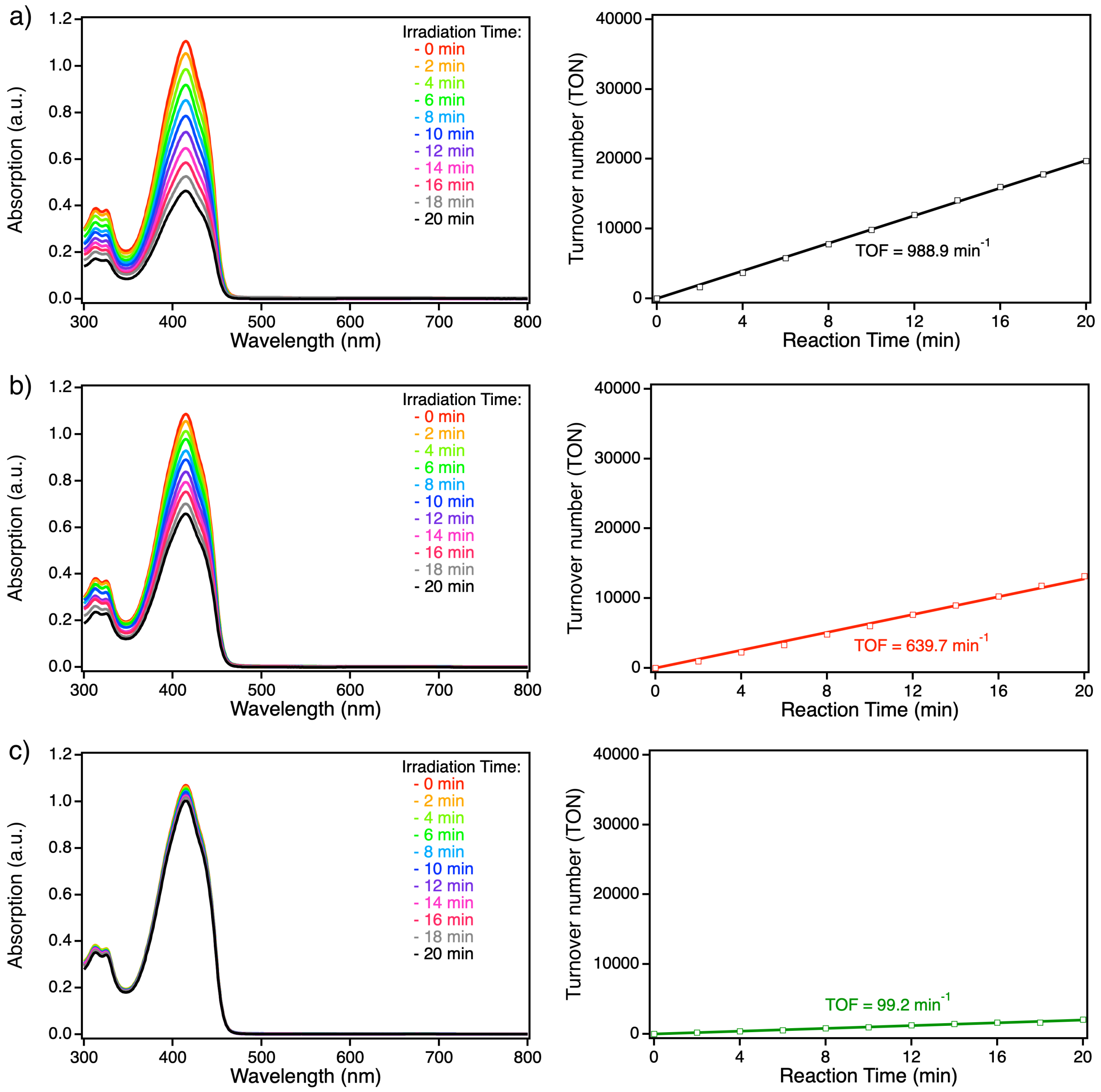

Fig. S1: UV Vis spectra for the photooxidation of DPBF with **H1-3** as hybrid photocatalyst employing either a 550 nm cut-on filter (a), a 700 nm bandpass filter (b) or a 550 nm bandpass filter (c) for irradiation. The amount of converted DPBF was determined using Labert Beers law and the extinction coefficinet of 23000 L mol^{-1} at λ = 415 nm. Turnover numbers (TON) were calculated from the amount of converted DPBF and the illuminated photosensitizer amount of 1.4 x 10^{-10} mol as determined from ICP-MS. Turnover frequencies (TOF) were obtained by linear regression from the plots of TON vs. reaction time in the linear regime before saturation effects become dominant.

ESI-2 Supplementary data for the photooxidation of DPBF with H2-3 using different irradiation wavelengths

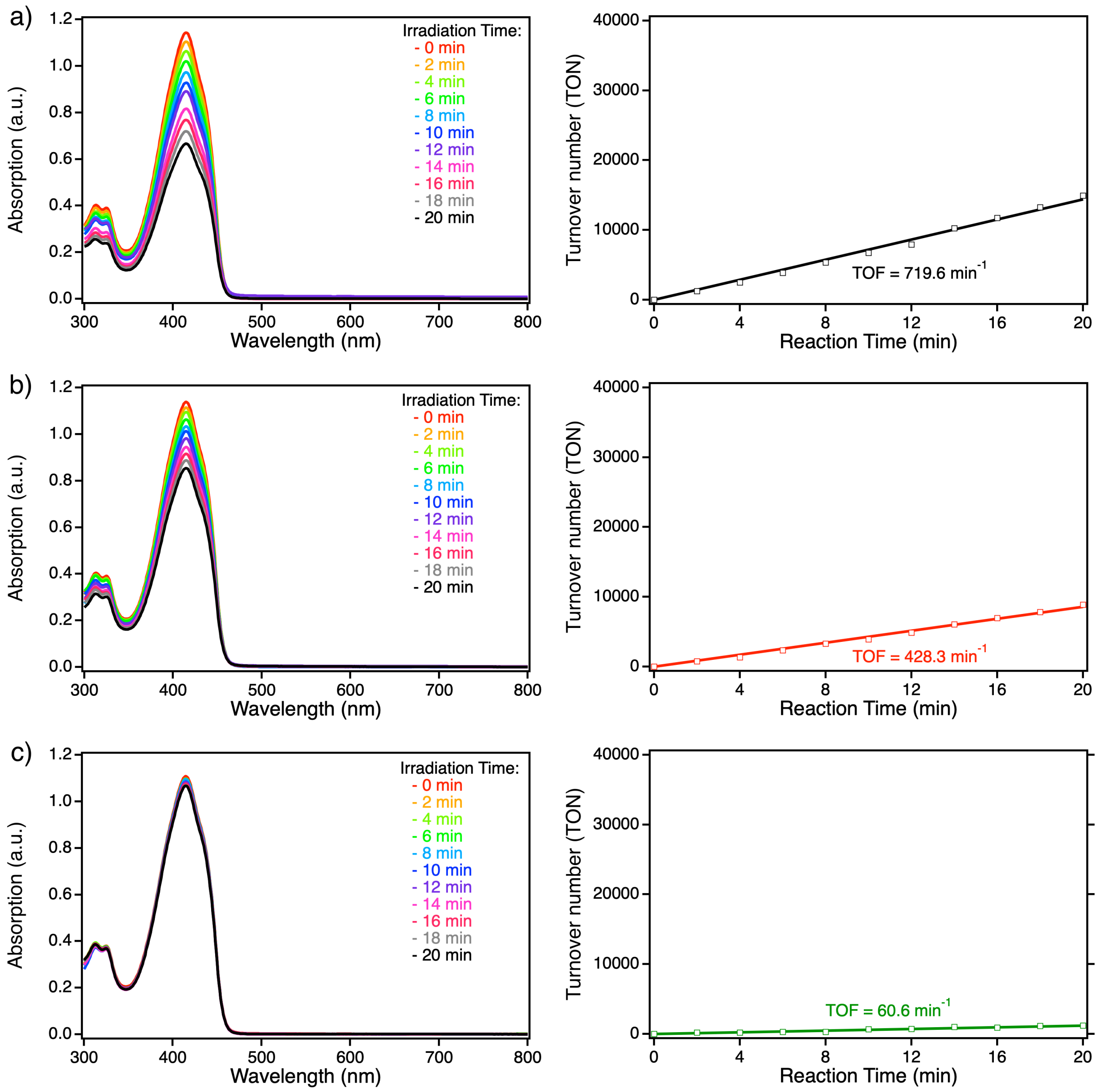

Fig. S2: UV Vis spectra for the photooxidation of DPBF with **H2-3** as hybrid photocatalyst employing either a 550 nm cut-on filter (a), a 700 nm bandpass filter (b) or a 550 nm bandpass filter (c) for irradiation. The amount of converted DPBF was determined using Labert Beers law and the extinction coefficinet of 23000 L mol^{-1} at λ = 415 nm. Turnover numbers (TON) were calculated from the amount of converted DPBF and the illuminated photosensitizer amount of 1.4 x 10^{-10} mol as determined from ICP-MS. Turnover frequencies (TOF) were obtained by linear regression from the plots of TON vs. reaction time in the linear regime before saturation effects become dominant.

ESI-3 Supplementary data for the photooxidation of DPBF with H1-4 using different irradiation wavelengths

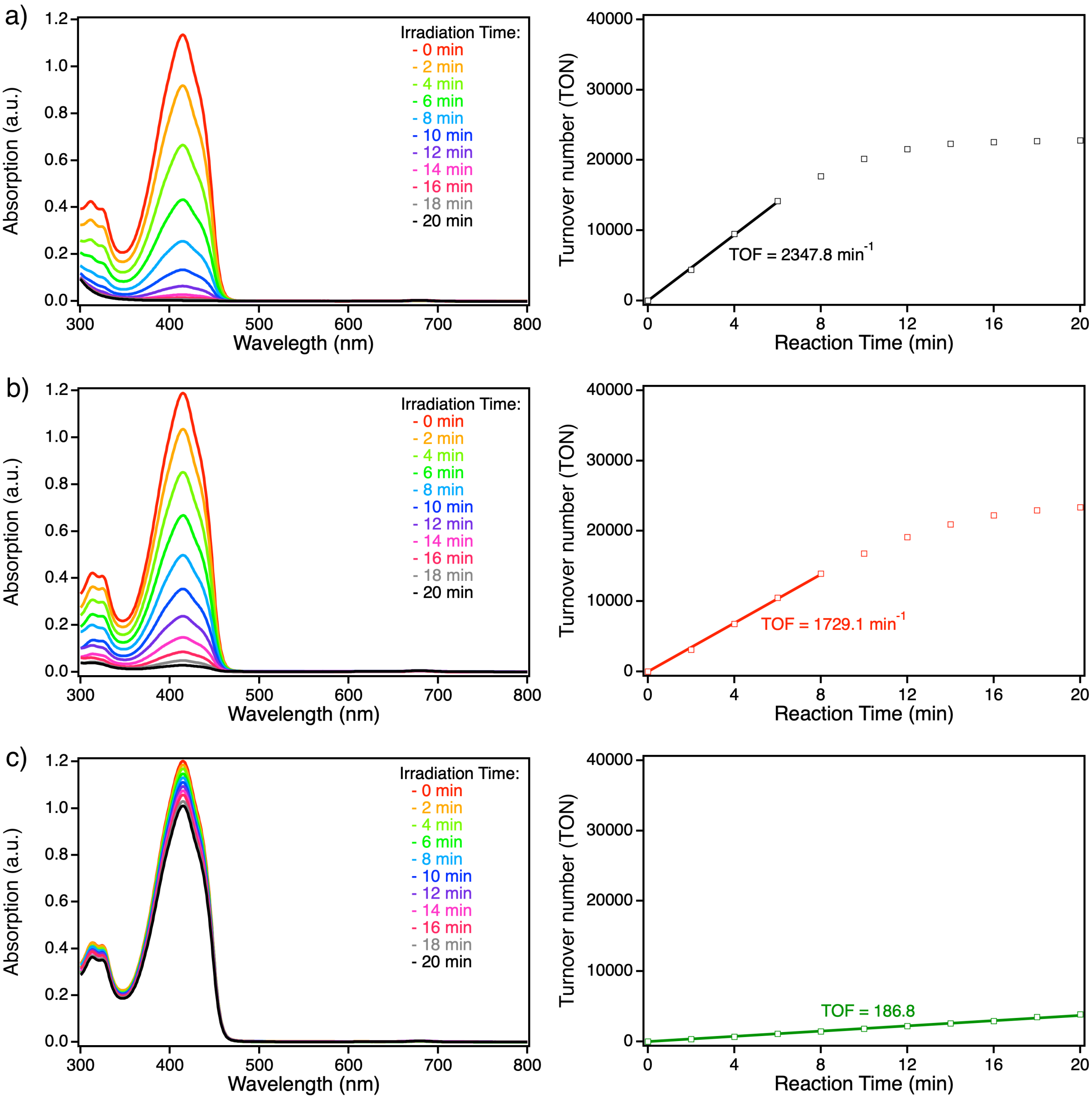

Fig. S3: UV Vis spectra for the photooxidation of DPBF with **H1-4** as hybrid photocatalyst employing either a 550 nm cut-on filter (a), a 700 nm bandpass filter (b) or a 550 nm bandpass filter (c) for irradiation. The amount of converted DPBF was determined using Labert Beers law and the extinction coefficinet of 23000 L mol^{-1} at λ = 415 nm. Turnover numbers (TON) were calculated from the amount of converted DPBF and the illuminated photosensitizer amount of 2.1 x 10^{-10} mol as determined from ICP-MS. Turnover frequencies (TOF) were obtained by linear regression from the plots of TON vs. reaction time in the linear regime before saturation effects become dominant.

ESI-4 Supplementary data for the photooxidation of DPBF with H2-4 using different irradiation wavelengths

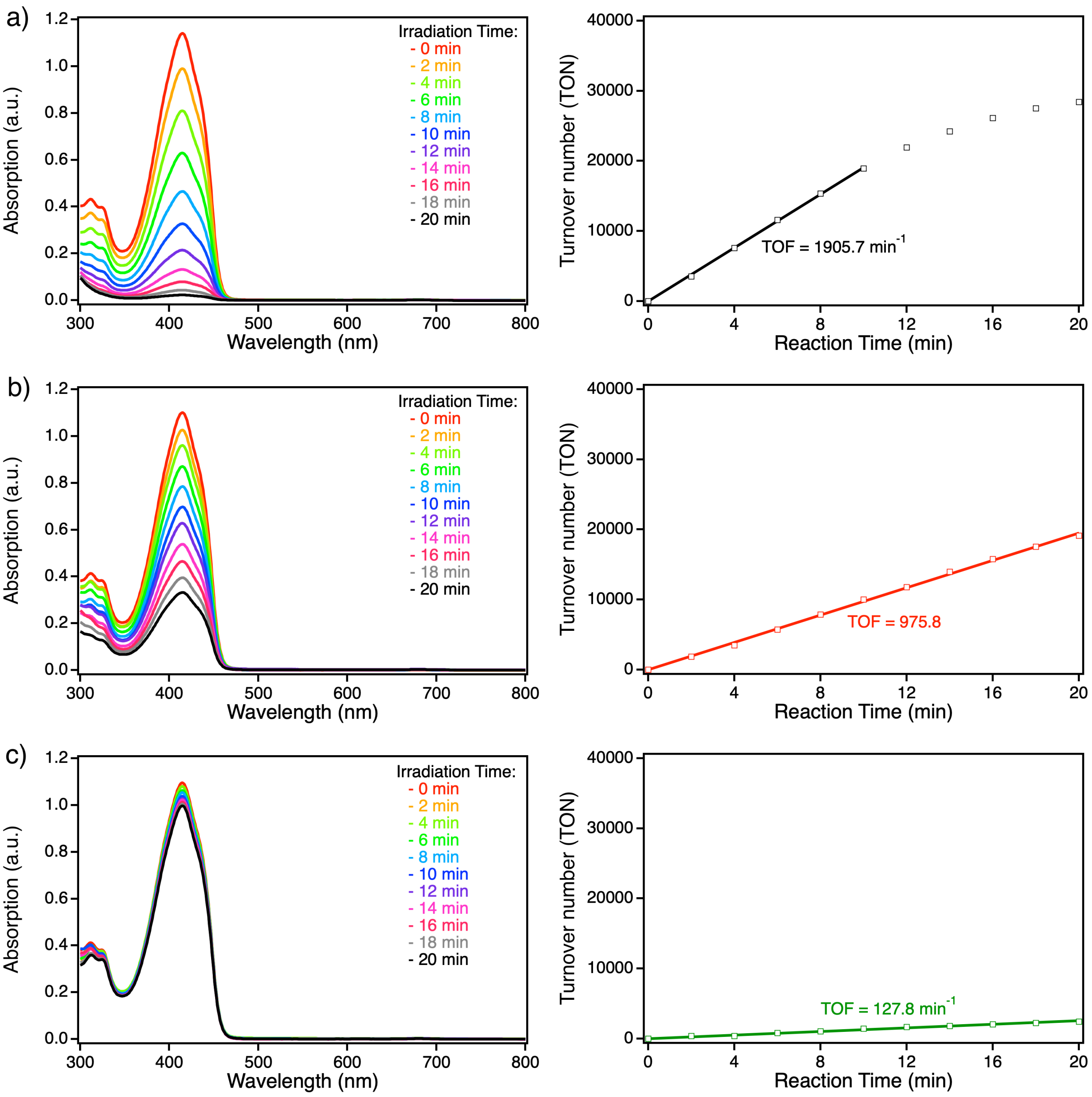

Fig. S4: UV Vis spectra for the photooxidation of DPBF with **H2-4** as hybrid photocatalyst employing either a 550 nm cut-on filter (a), a 700 nm bandpass filter (b) or a 550 nm bandpass filter (c) for irradiation. The amount of converted DPBF was determined using Labert Beers law and the extinction coefficinet of 23000 L mol^{-1} at λ = 415 nm. Turnover numbers (TON) were calculated from the amount of converted DPBF and the illuminated photosensitizer amount of 1.8 x 10^{-10} mol as determined from ICP-MS. Turnover frequencies (TOF) were obtained by linear regression from the plots of TON vs. reaction time in the linear regime before saturation effects become dominant.

ESI-5 Supplementary data for the photooxidation of DPBF with H1-5 using different irradiation wavelengths

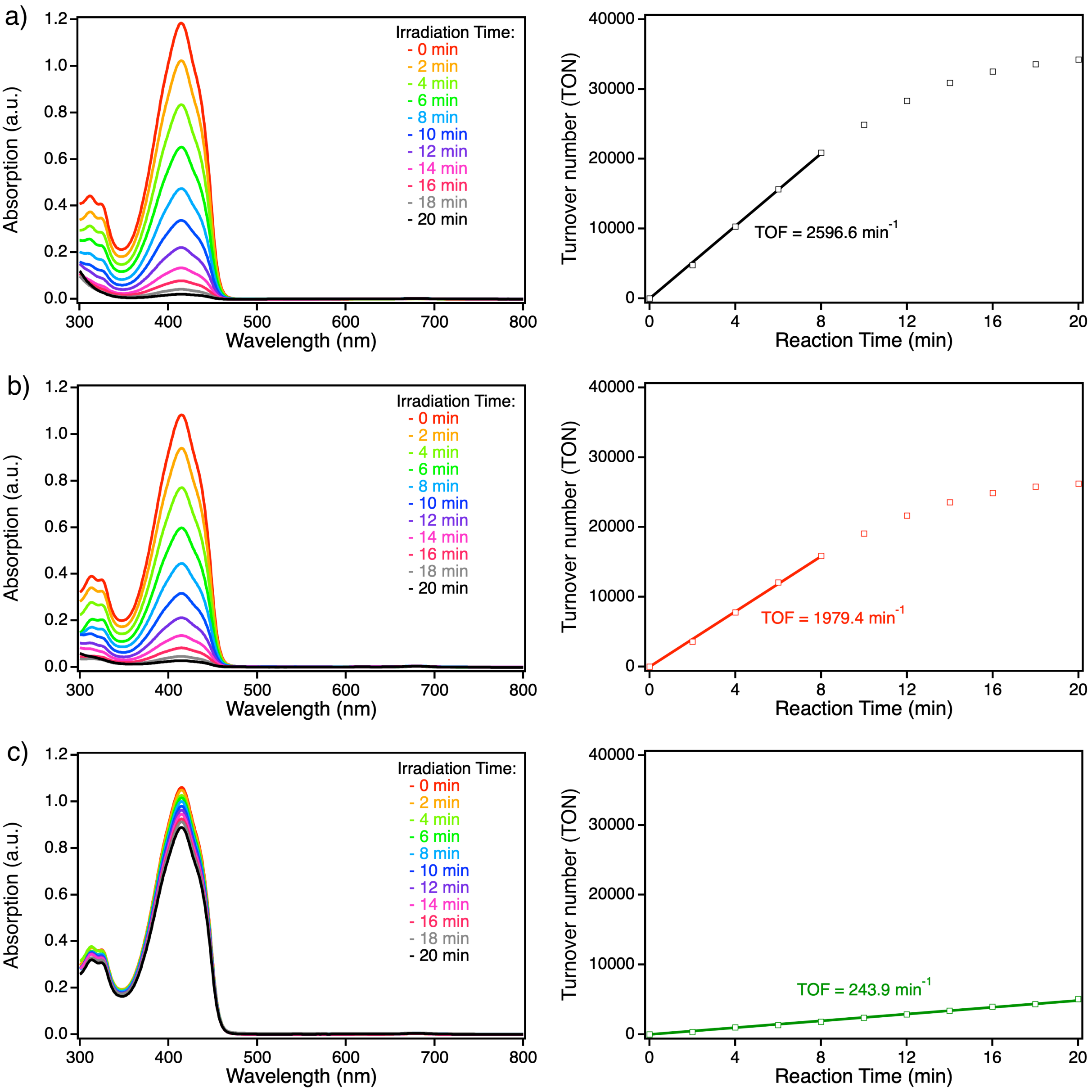

Fig. S5: UV Vis spectra for the photooxidation of DPBF with **H1-5** as hybrid photocatalyst employing either a 550 nm cut-on filter (a), a 700 nm bandpass filter (b) or a 550 nm bandpass filter (c) for irradiation. The amount of converted DPBF was determined using Labert Beers law and the extinction coefficinet of 23000 L mol^{-1} at λ = 415 nm. Turnover numbers (TON) were calculated from the amount of converted DPBF and the illuminated photosensitizer amount of 1.5 x 10^{-10} mol as determined from ICP-MS. Turnover frequencies (TOF) were obtained by linear regression from the plots of TON vs. reaction time in the linear regime before saturation effects become dominant.

ESI-6 Supplementary data for the photooxidation of DPBF with H2-5 using different irradiation wavelengths

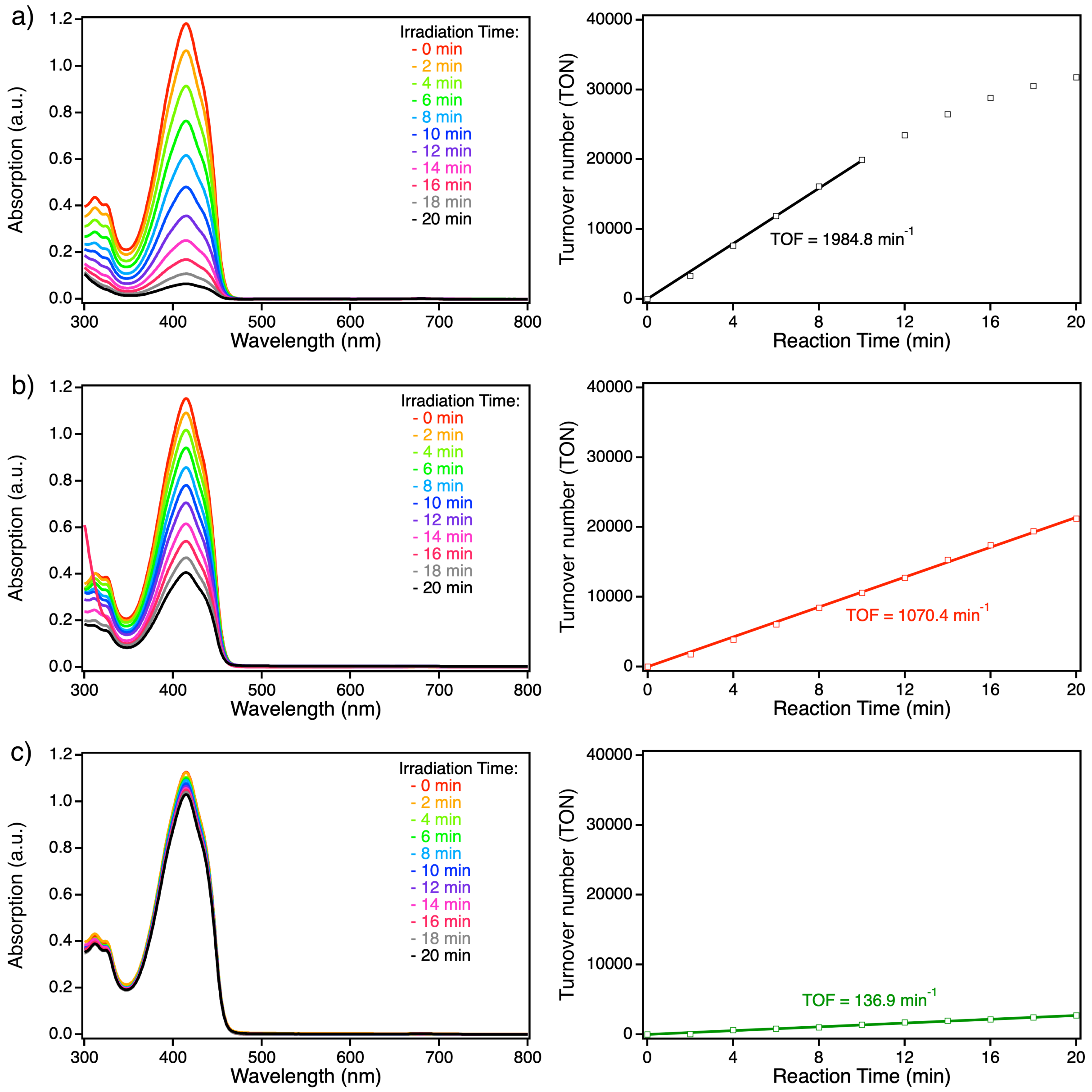

Fig. S6: UV Vis spectra for the photooxidation of DPBF with **H2-5** as hybrid photocatalyst employing either a 550 nm cut-on filter (a), a 700 nm bandpass filter (b) or a 550 nm bandpass filter (c) for irradiation. The amount of converted DPBF was determined using Labert Beers law and the extinction coefficinet of 23000 L mol^{-1} at λ = 415 nm. Turnover numbers (TON) were calculated from the amount of converted DPBF and the illuminated photosensitizer amount of 1.5 x 10^{-10} mol as determined from ICP-MS. Turnover frequencies (TOF) were obtained by linear regression from the plots of TON vs. reaction time in the linear regime before saturation effects become dominant.

ESI-7 Supplementary data for the photooxidation of DPBF with H1-6 using different irradiation wavelengths

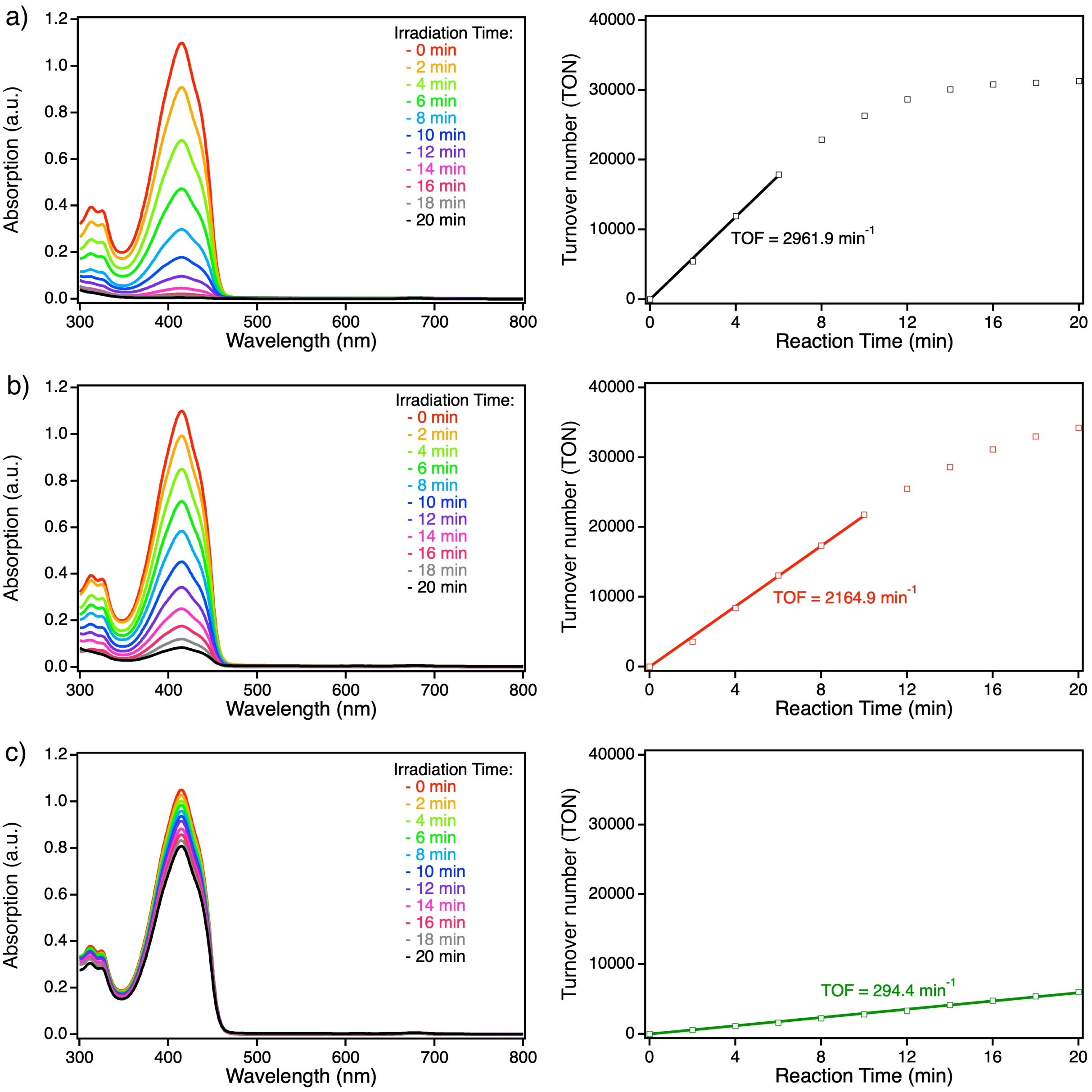

Fig. S7: UV Vis spectra for the photooxidation of DPBF with **H1-6** as hybrid photocatalyst employing either a 550 nm cut-on filter (a), a 700 nm bandpass filter (b) or a 550 nm bandpass filter (c) for irradiation. The amount of converted DPBF was determined using Labert Beers law and the extinction coefficinet of 23000 L mol^{-1} at λ = 415 nm. Turnover numbers (TON) were calculated from the amount of converted DPBF and the illuminated photosensitizer amount of 1.5 x 10^{-10} mol as determined from ICP-MS. Turnover frequencies (TOF) were obtained by linear regression from the plots of TON vs. reaction time in the linear regime before saturation effects become dominant.

ESI-8 Supplementary data for the photooxidation of DPBF with H2-6 using different irradiation wavelengths

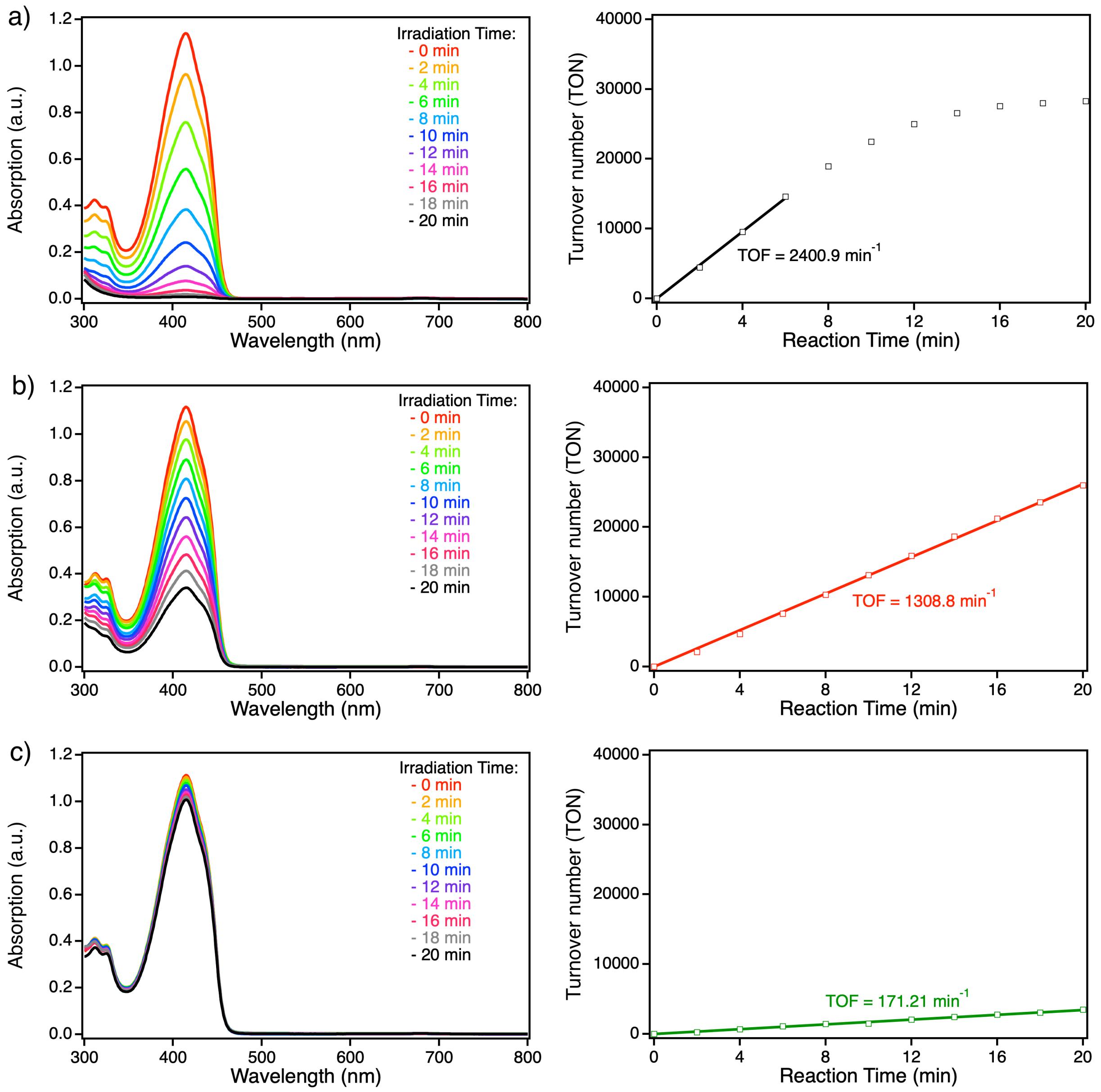

Fig. S8: UV Vis spectra for the photooxidation of DPBF with **H2-6** as hybrid photocatalyst employing either a 550 nm cut-on filter (a), a 700 nm bandpass filter (b) or a 550 nm bandpass filter (c) for irradiation. The amount of converted DPBF was determined using Labert Beers law and the extinction coefficinet of 23000 L mol^{-1} at λ = 415 nm. Turnover numbers (TON) were calculated from the amount of converted DPBF and the illuminated photosensitizer amount of 1.7 x 10^{-10} mol as determined from ICP-MS. Turnover frequencies (TOF) were obtained by linear regression from the plots of TON vs. reaction time in the linear regime before saturation effects become dominant.

ESI-9 Supplementary data for the photooxidation of DPBF with H1-7 using different irradiation wavelengths

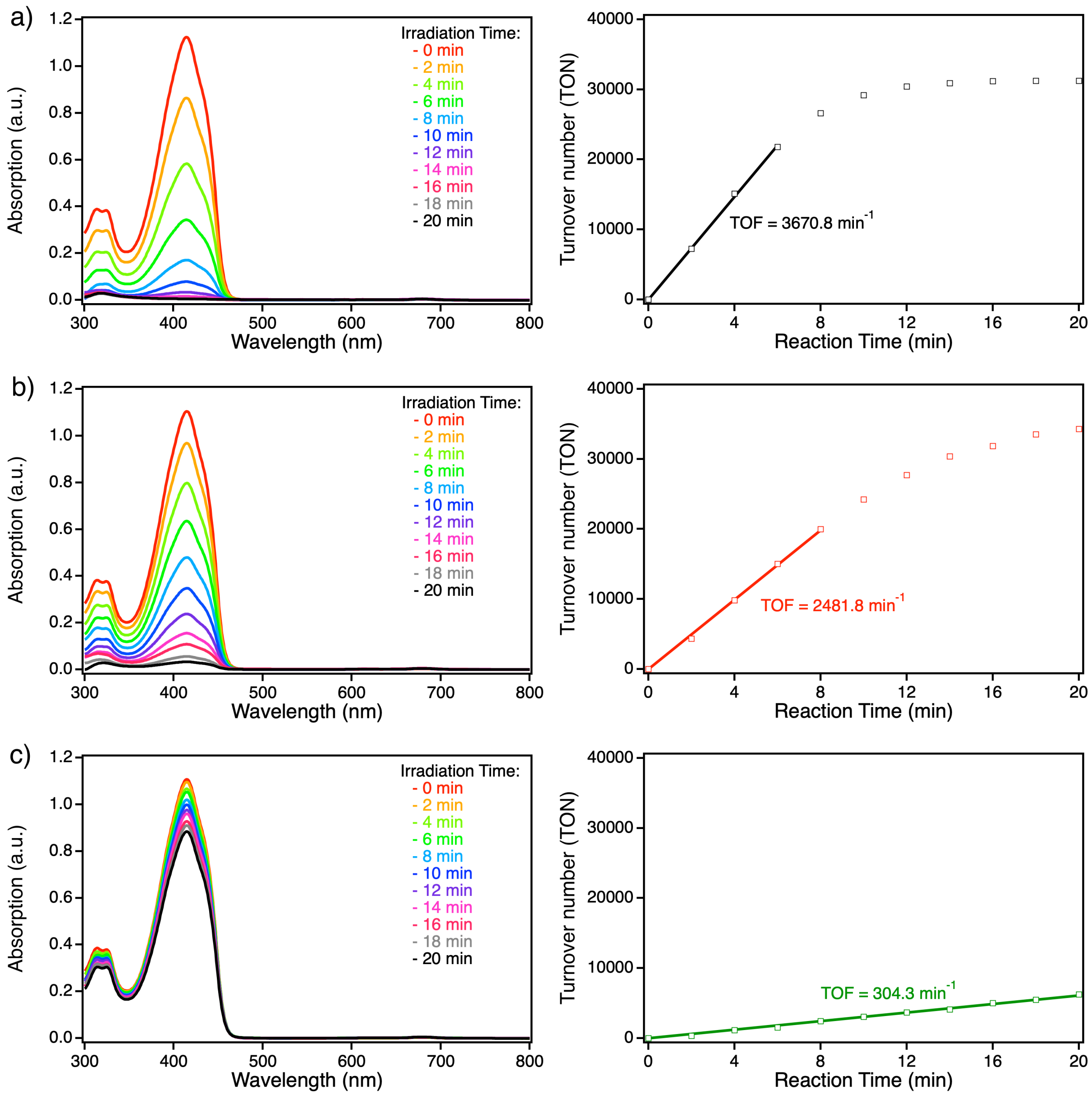

Fig. S9: UV Vis spectra for the photooxidation of DPBF with **H1-7** as hybrid photocatalyst employing either a 550 nm cut-on filter (a), a 700 nm bandpass filter (b) or a 550 nm bandpass filter (c) for irradiation. The amount of converted DPBF was determined using Labert Beers law and the extinction coefficinet of 23000 L mol^{-1} at λ = 415 nm. Turnover numbers (TON) were calculated from the amount of converted DPBF and the illuminated photosensitizer amount of 1.6 x 10^{-10} mol as determined from ICP-MS. Turnover frequencies (TOF) were obtained by linear regression from the plots of TON vs. reaction time in the linear regime before saturation effects become dominant.

ESI-10 Supplementary data for the photooxidation of DPBF with H2-7 using different irradiation wavelengths

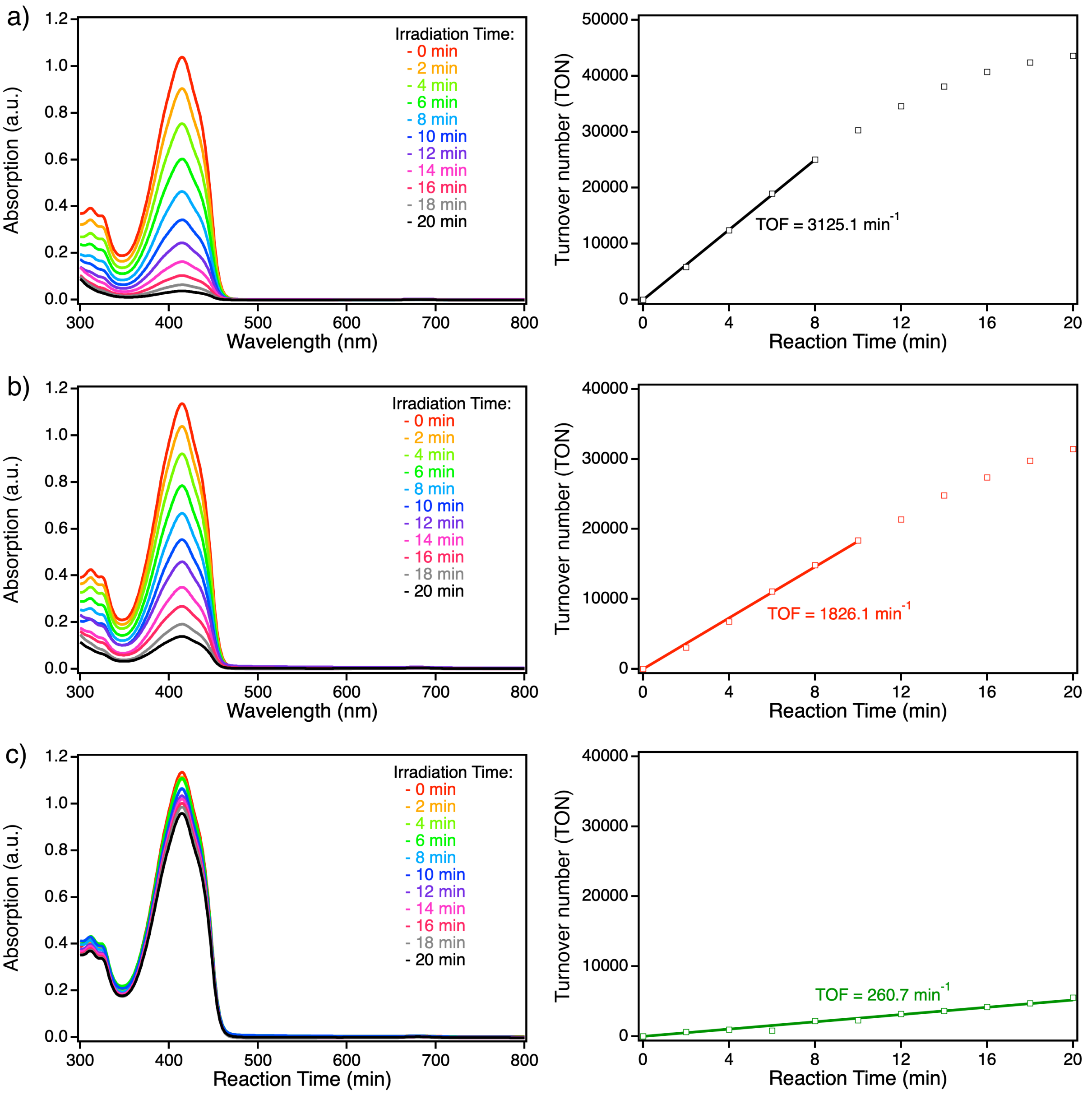

Fig. S10: UV Vis spectra for the photooxidation of DPBF with **H2-7** as hybrid photocatalyst employing either a 550 nm cut-on filter (a), a 700 nm bandpass filter (b) or a 550 nm bandpass filter (c) for irradiation. The amount of converted DPBF was determined using Labert Beers law and the extinction coefficinet of 23000 L mol^{-1} at λ = 415 nm. Turnover numbers (TON) were calculated from the amount of converted DPBF and the illuminated photosensitizer amount of 1.4×10^{-10} mol as determined from ICP-MS. Turnover frequencies (TOF) were obtained by linear regression from the plots of TON vs. reaction time in the linear regime before saturation effects become dominant.

ESI-11 Supplementary data for the photooxidation of DPBF with H1-8 using different irradiation wavelengths

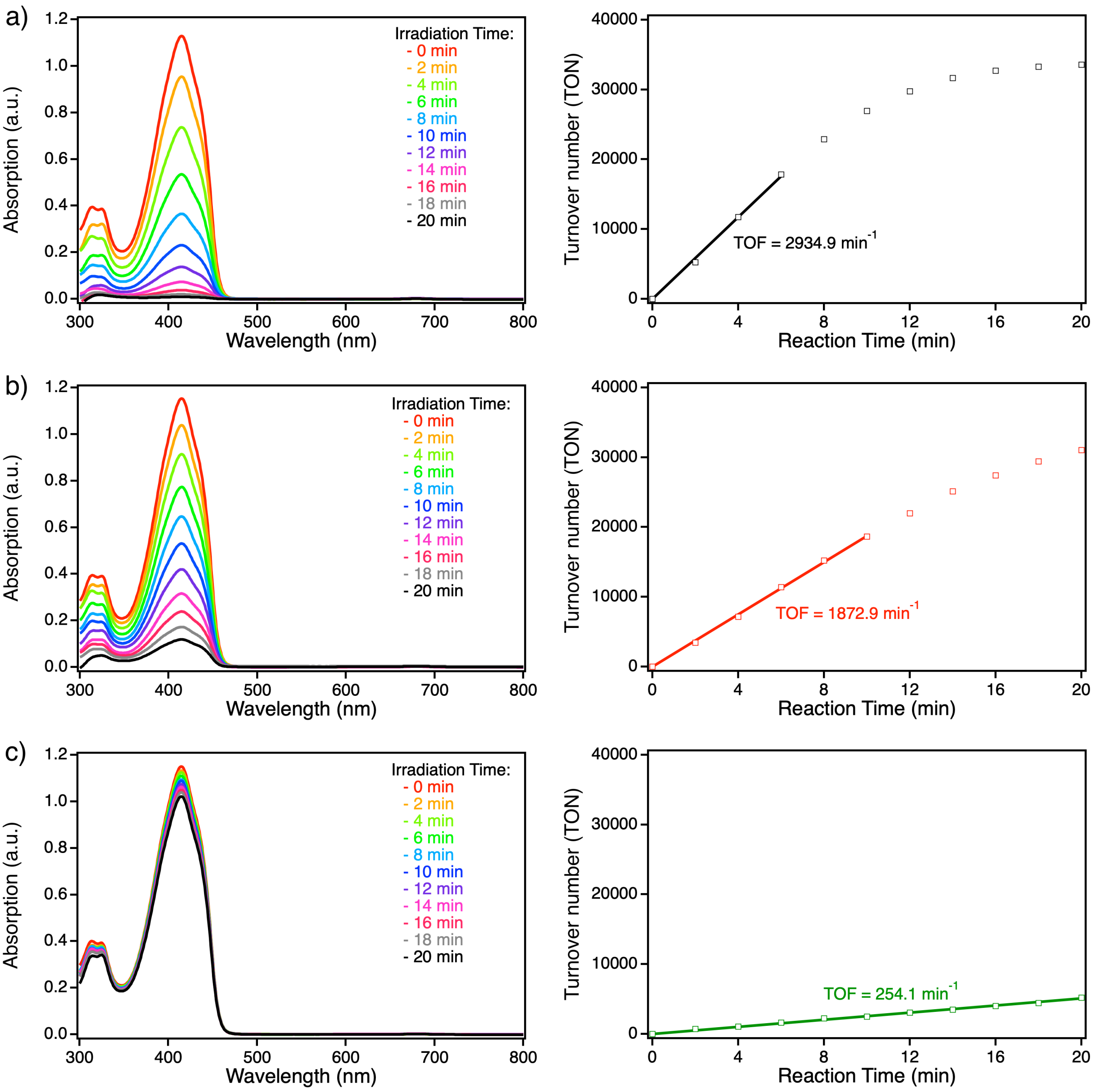

Fig. S11: UV Vis spectra for the photooxidation of DPBF with **H1-8** as hybrid photocatalyst employing either a 550 nm cut-on filter (a), a 700 nm bandpass filter (b) or a 550 nm bandpass filter (c) for irradiation. The amount of converted DPBF was determined using Labert Beers law and the extinction coefficinet of 23000 L mol^{-1} at λ = 415 nm. Turnover numbers (TON) were calculated from the amount of converted DPBF and the illuminated photosensitizer amount of 1.3 x 10^{-10} mol as determined from ICP-MS. Turnover frequencies (TOF) were obtained by linear regression from the plots of TON vs. reaction time in the linear regime before saturation effects become dominant.

ESI-12 Supplementary data for the photooxidation of DPBF with H2-8 using different irradiation wavelengths

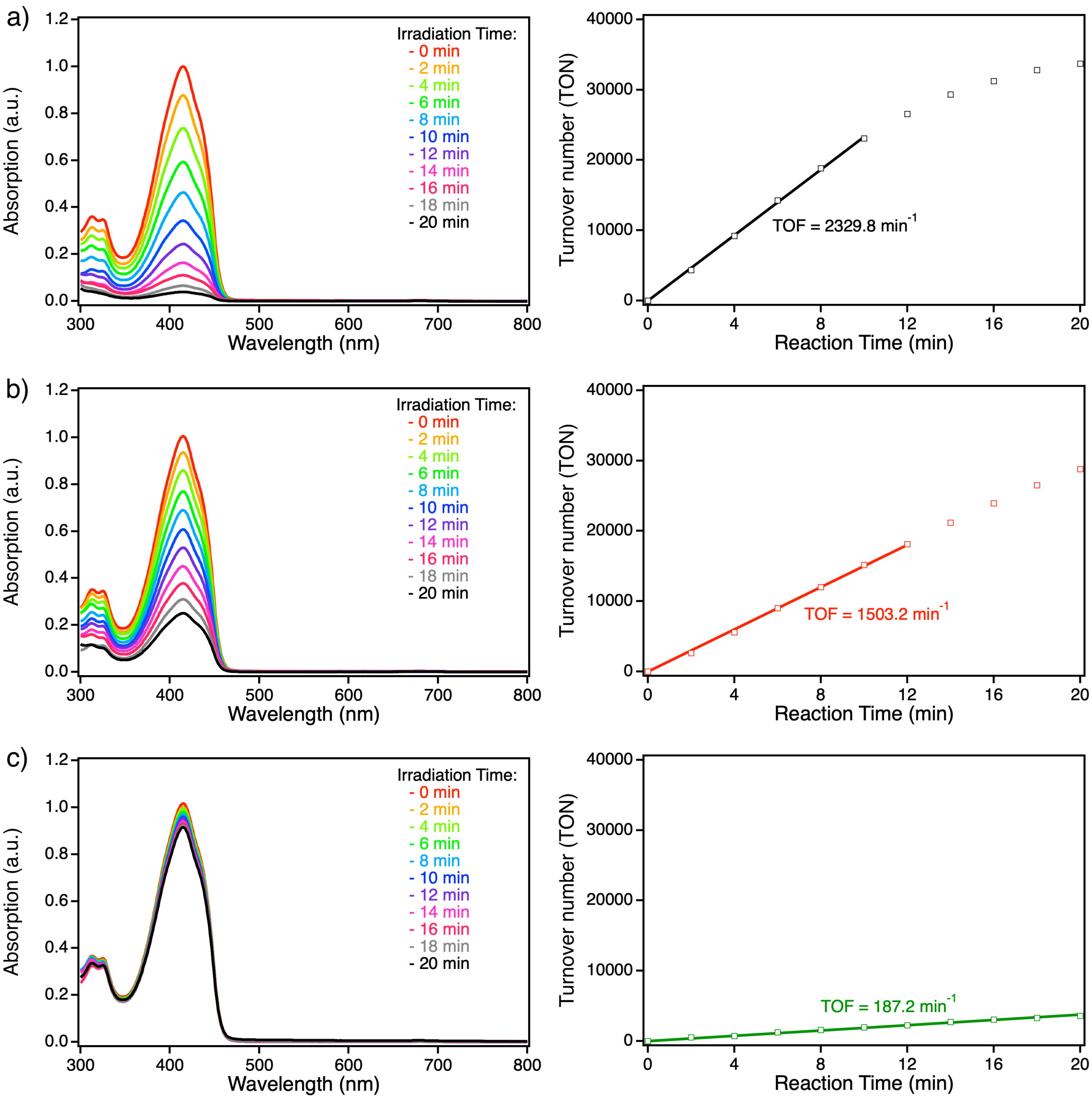

Fig. S12: UV Vis spectra for the photooxidation of DPBF with **H2-8** as hybrid photocatalyst employing either a 550 nm cut-on filter (a), a 700 nm bandpass filter (b) or a 550 nm bandpass filter (c) for irradiation. The amount of converted DPBF was determined using Labert Beers law and the extinction coefficinet of 23000 L mol^{-1} at λ = 415 nm. Turnover numbers (TON) were calculated from the amount of converted DPBF and the illuminated photosensitizer amount of 1.2 x 10^{-10} mol as determined from ICP-MS. Turnover frequencies (TOF) were obtained by linear regression from the plots of TON vs. reaction time in the linear regime before saturation effects become dominant.

ESI-13 Supplementary data for the photooxidation of DPBF with H1-9 using different irradiation wavelengths

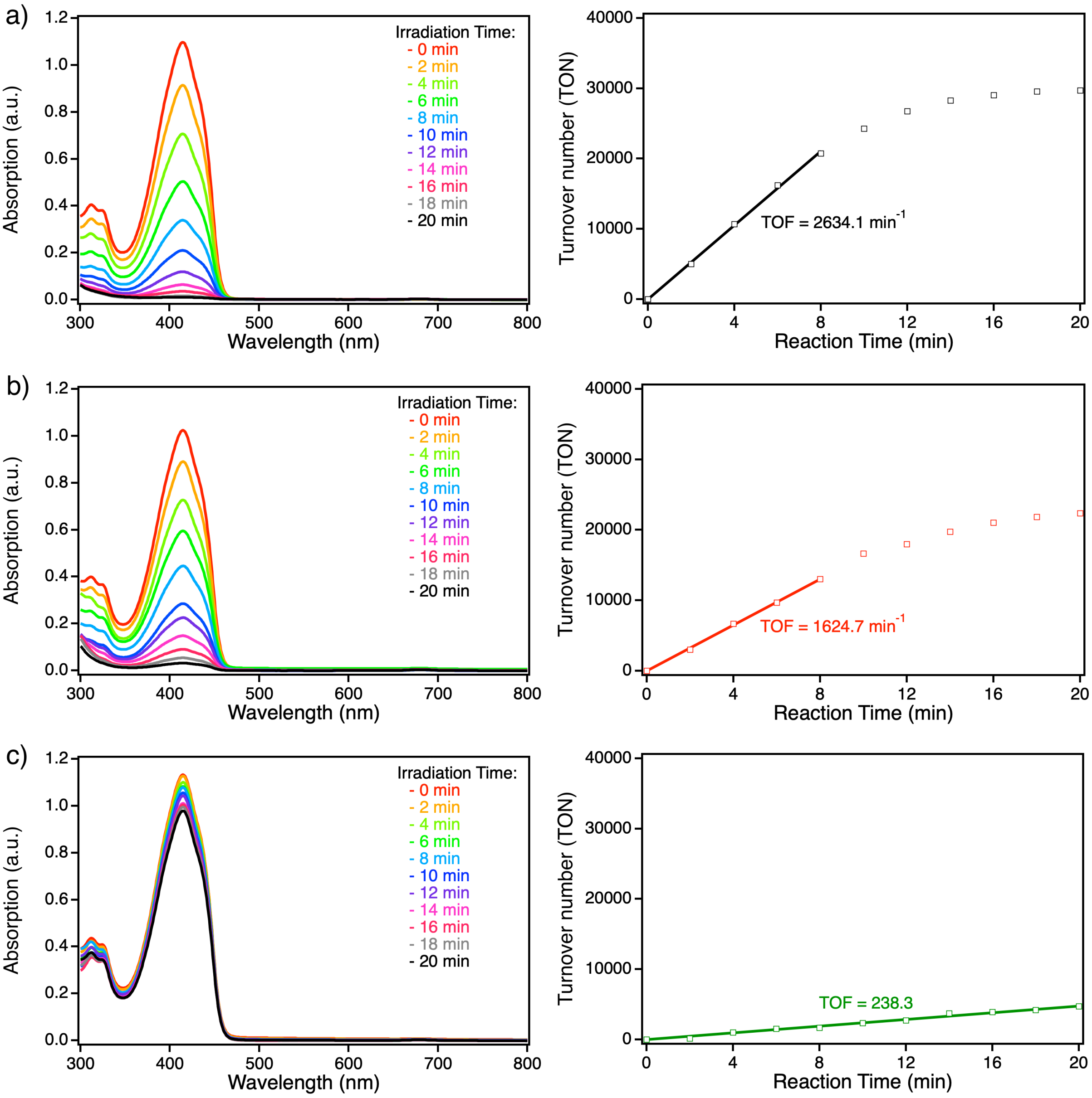

Fig. S13: UV Vis spectra for the photooxidation of DPBF with **H1-9** as hybrid photocatalyst employing either a 550 nm cut-on filter (a), a 700 nm bandpass filter (b) or a 550 nm bandpass filter (c) for irradiation. The amount of converted DPBF was determined using Labert Beers law and the extinction coefficinet of 23000 L mol^{-1} at λ = 415 nm. Turnover numbers (TON) were calculated from the amount of converted DPBF and the illuminated photosensitizer amount of 1.4 x 10^{-10} mol as determined from ICP-MS. Turnover frequencies (TOF) were obtained by linear regression from the plots of TON vs. reaction time in the linear regime before saturation effects become dominant.

ESI-14 Supplementary data for the photooxidation of DPBF with H2-9 using different irradiation wavelengths

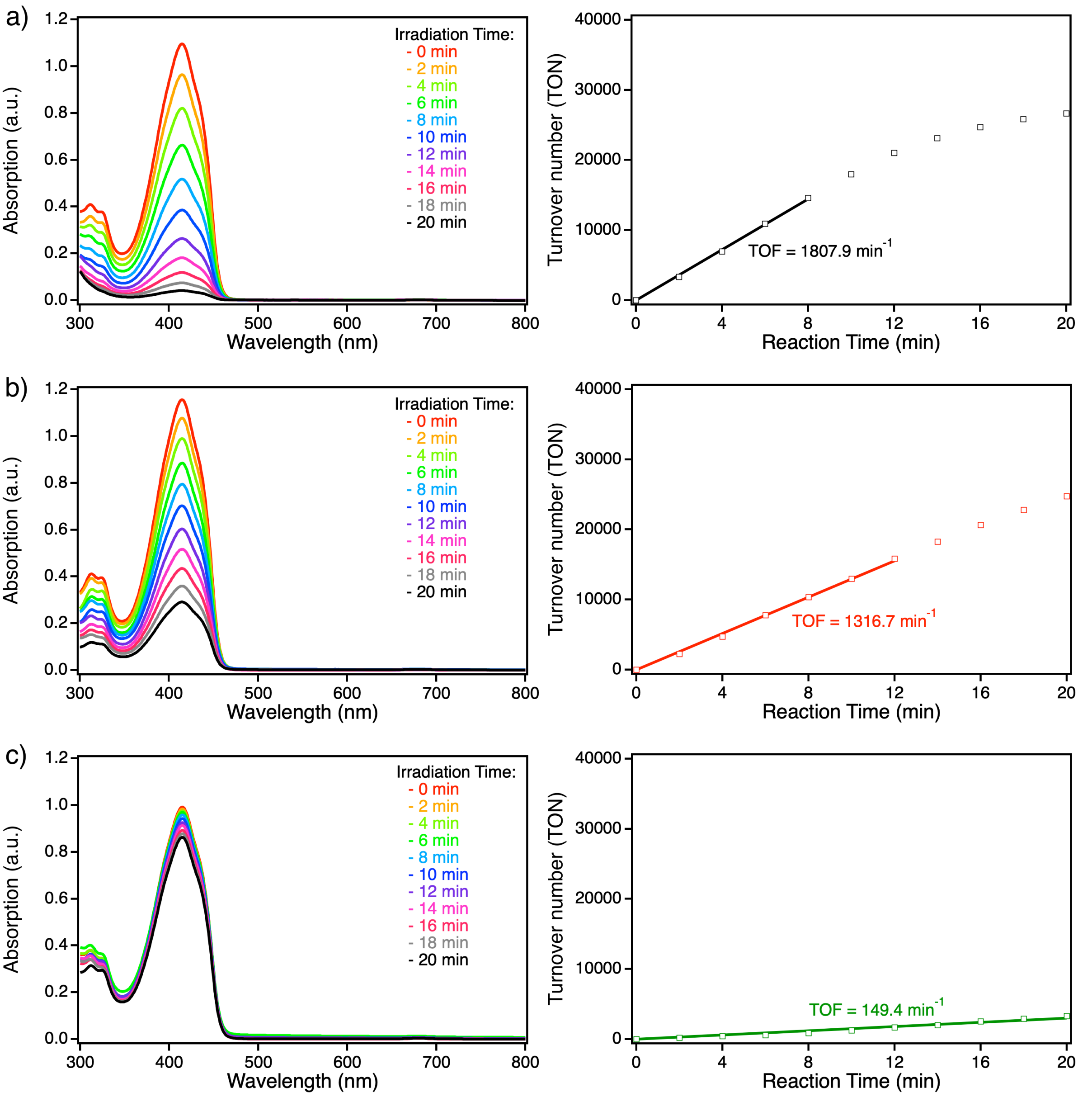

Fig. S14: UV Vis spectra for the photooxidation of DPBF with **H2-9** as hybrid photocatalyst employing either a 550 nm cut-on filter (a), a 700 nm bandpass filter (b) or a 550 nm bandpass filter (c) for irradiation. The amount of converted DPBF was determined using Labert Beers law and the extinction coefficinet of 23000 L mol^{-1} at λ = 415 nm. Turnover numbers (TON) were calculated from the amount of converted DPBF and the illuminated photosensitizer amount of 1.7 x 10^{-10} mol as determined from ICP-MS. Turnover frequencies (TOF) were obtained by linear regression from the plots of TON vs. reaction time in the linear regime before saturation effects become dominant.

ESI-15 Supplementary data for the photooxidation of DPBF with H1-10 using different irradiation wavelengths

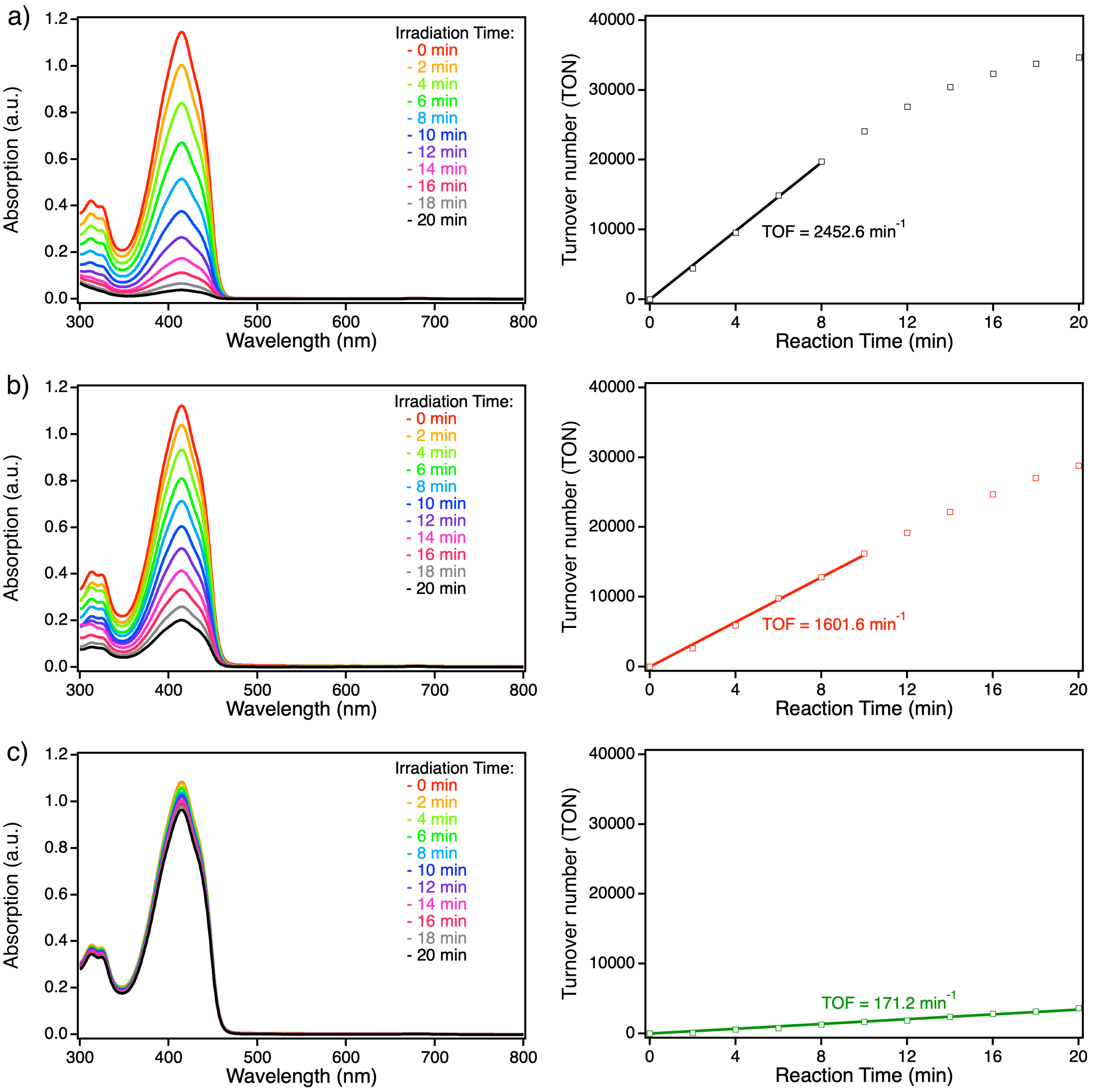

Fig. S15: UV Vis spectra for the photooxidation of DPBF with **H1-10** as hybrid photocatalyst employing either a 550 nm cut-on filter (a), a 700 nm bandpass filter (b) or a 550 nm bandpass filter (c) for irradiation. The amount of converted DPBF was determined using Labert Beers law and the extinction coefficinet of 23000 L mol^{-1} at λ = 415 nm. Turnover numbers (TON) were calculated from the amount of converted DPBF and the illuminated photosensitizer amount of 1.4 x 10^{-10} mol as determined from ICP-MS. Turnover frequencies (TOF) were obtained by linear regression from the plots of TON vs. reaction time in the linear regime before saturation effects become dominant.

ESI-16 Supplementary data for the photooxidation of DPBF with H2-10 using different irradiation wavelengths

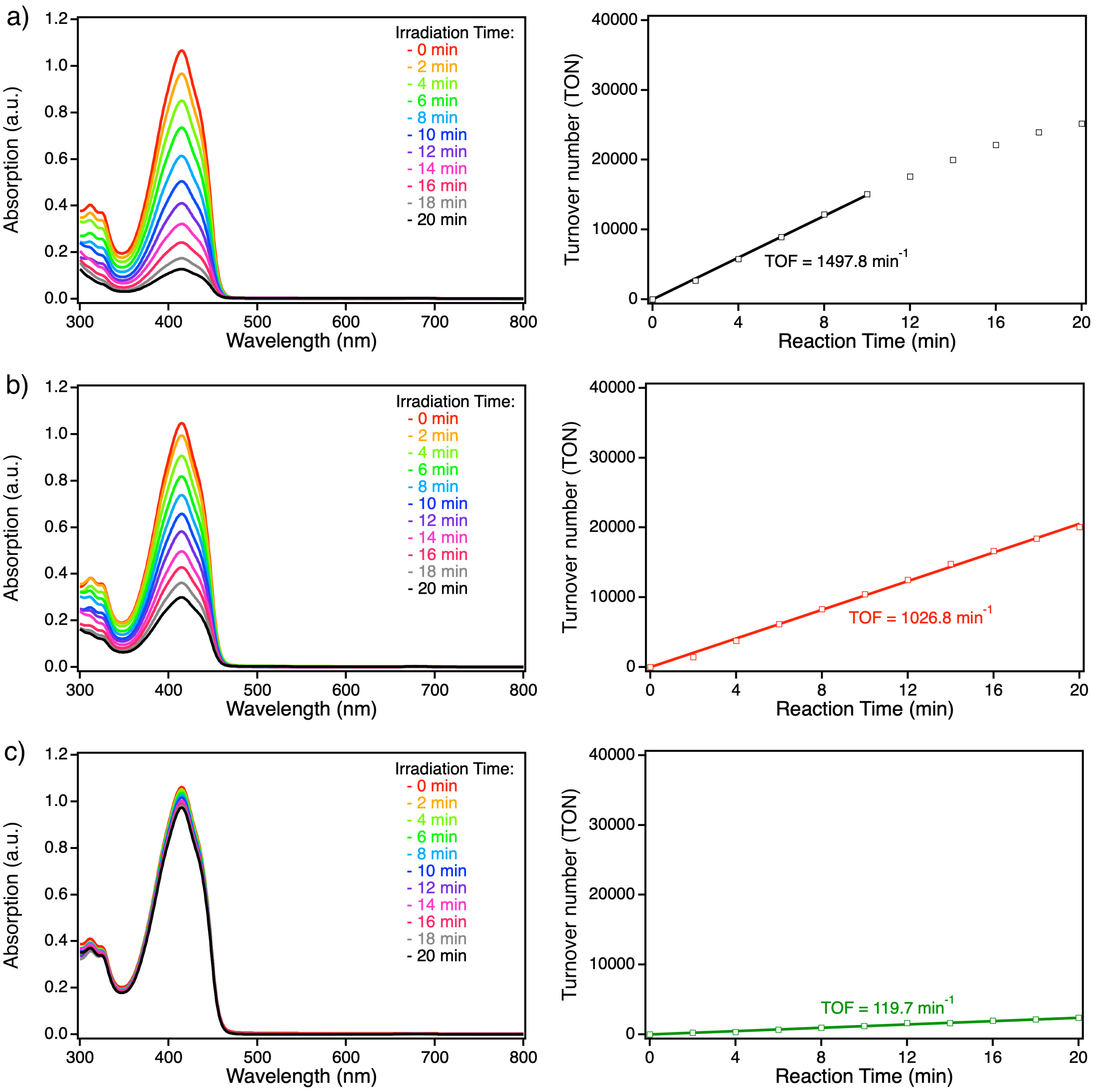

Fig. S16: UV Vis spectra for the photooxidation of DPBF with **H2-10** as hybrid photocatalyst employing either a 550 nm cut-on filter (a), a 700 nm bandpass filter (b) or a 550 nm bandpass filter (c) for irradiation. The amount of converted DPBF was determined using Labert Beers law and the extinction coefficinet of 23000 L mol^{-1} at λ = 415 nm. Turnover numbers (TON) were calculated from the amount of converted DPBF and the illuminated photosensitizer amount of 1.6 x 10^{-10} mol as determined from ICP-MS. Turnover frequencies (TOF) were obtained by linear regression from the plots of TON vs. reaction time in the linear regime before saturation effects become dominant.

ESI-17 Supplementary data for the photooxidation of DPBF with H1-11 using different irradiation wavelengths

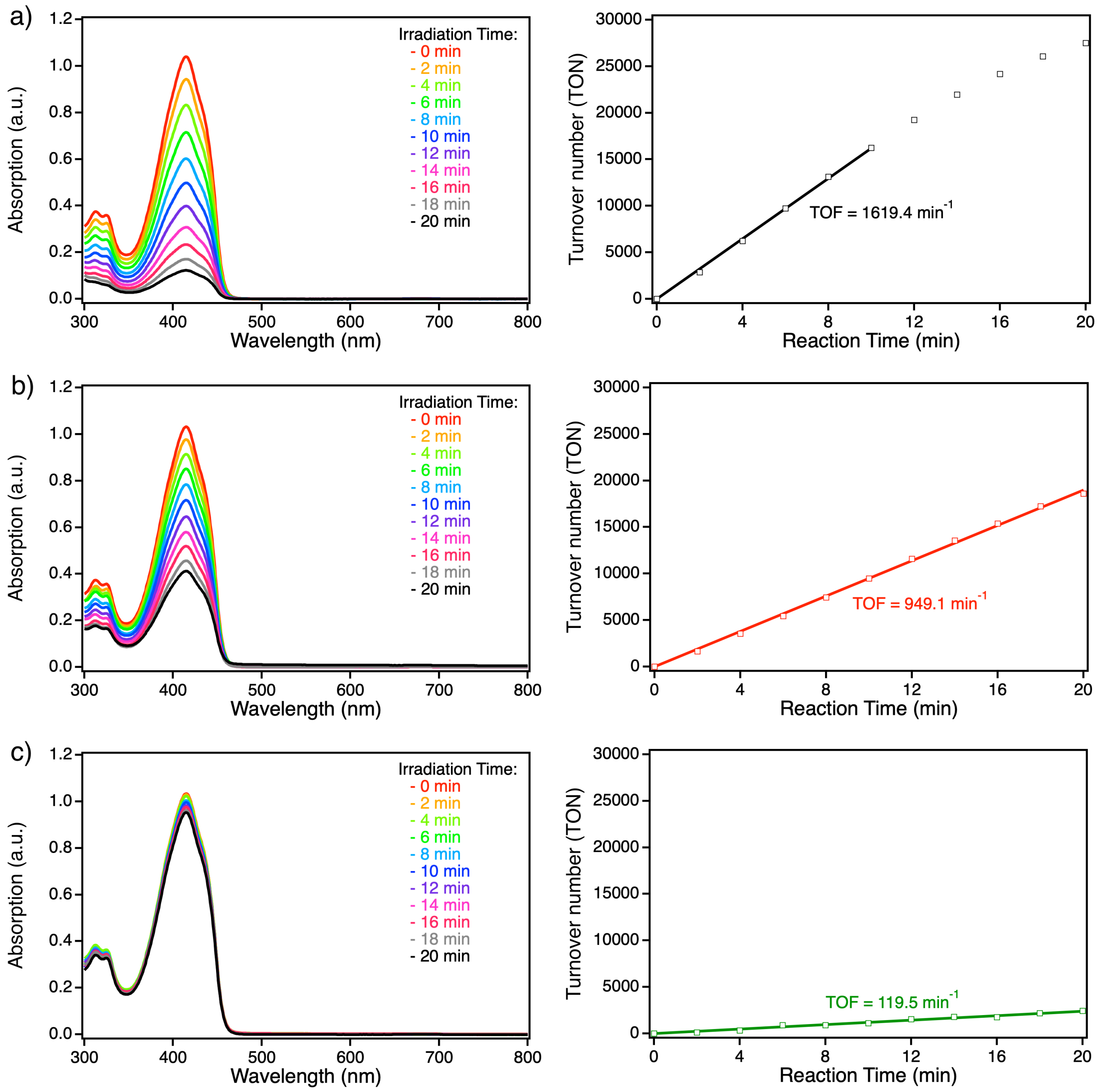

Fig. S17: UV Vis spectra for the photooxidation of DPBF with **H1-11** as hybrid photocatalyst employing either a 550 nm cut-on filter (a), a 700 nm bandpass filter (b) or a 550 nm bandpass filter (c) for irradiation. The amount of converted DPBF was determined using Labert Beers law and the extinction coefficinet of 23000 L mol^{-1} at λ = 415 nm. Turnover numbers (TON) were calculated from the amount of converted DPBF and the illuminated photosensitizer amount of 1.5 x 10^{-10} mol as determined from ICP-MS. Turnover frequencies (TOF) were obtained by linear regression from the plots of TON vs. reaction time in the linear regime before saturation effects become dominant.

ESI-18 Supplementary data for the photooxidation of DPBF with H2-11 using different irradiation wavelengths

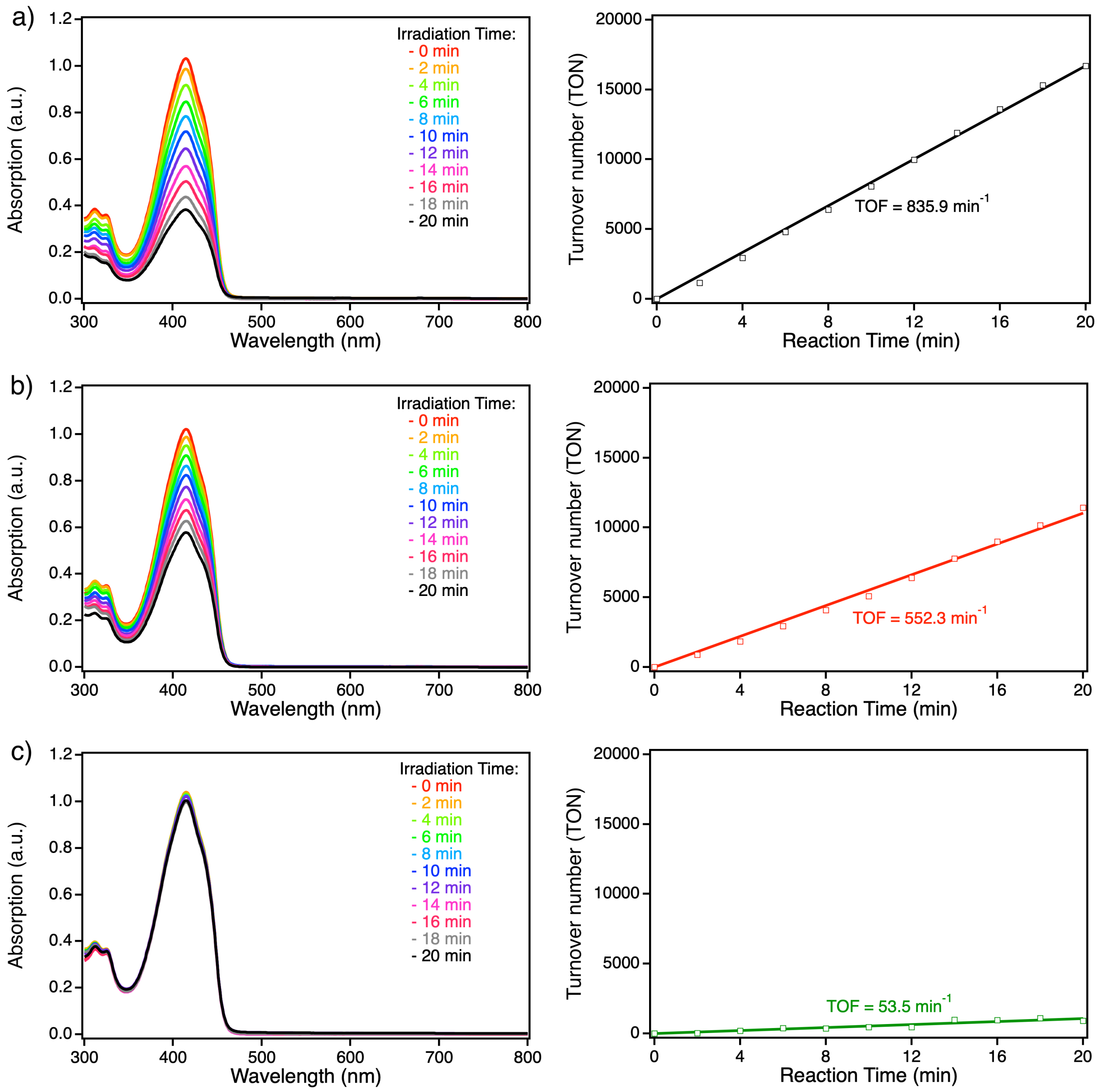

Fig. S18: UV Vis spectra for the photooxidation of DPBF with **H2-11** as hybrid photocatalyst employing either a 550 nm cut-on filter (a), a 700 nm bandpass filter (b) or a 550 nm bandpass filter (c) for irradiation. The amount of converted DPBF was determined using Labert Beers law and the extinction coefficinet of 23000 L mol^{-1} at λ = 415 nm. Turnover numbers (TON) were calculated from the amount of converted DPBF and the illuminated photosensitizer amount of 1.7 x 10^{-10} mol as determined from ICP-MS. Turnover frequencies (TOF) were obtained by linear regression from the plots of TON vs. reaction time in the linear regime before saturation effects become dominant.

ESI-19 Supplementary data fot the synthesis of the ZnPc derivatives 1 and 2

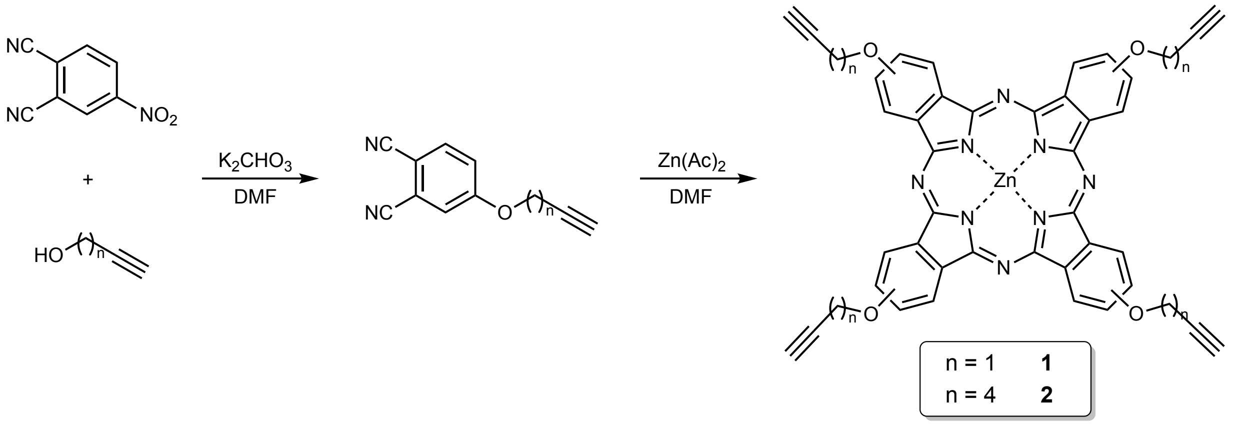

Scheme S1: Synthesis scheme for the photosensitizers *2,9,16,23-tetrakis(2-propyn-1-yloxy)phthalocyanine zinc(II)* (**1**) and *2,9,16,23-tetrakis(5-hexyn-1-yloxy) phthalocyanine zinc(II)* (**2**).

ESI-20 Supplementary data fot the synthesis of azidoalkylthioacetates 3 - 11

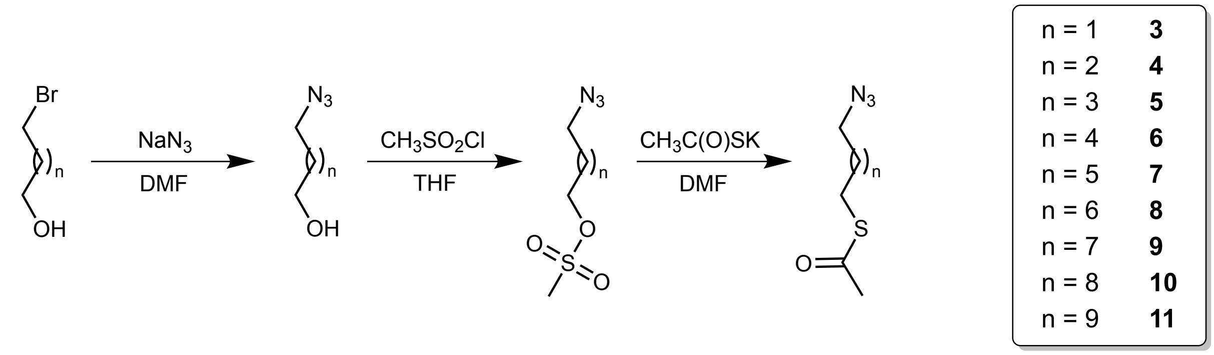

Scheme S2: General synthesis scheme for the azidoalkylthioacetate SAM precursors **3-11** from the corresponding *n*-bromo-1-alkanol via *n*-azidoalkan-1-ol and *n*-azidoalkyl methanesulfonate.
